# Modeling HIV-1 Within-Host Dynamics After Passive Infusion of the Broadly Neutralizing Antibody VRC01

**DOI:** 10.3389/fimmu.2021.710012

**Published:** 2021-08-31

**Authors:** E. Fabian Cardozo-Ojeda, Alan S. Perelson

**Affiliations:** ^1^Vaccine and Infectious Disease Division, Fred Hutchinson Cancer Research Center, Seattle, WA, United States; ^2^Theoretical Biology and Biophysics, Los Alamos National Laboratory, Los Alamos, NM, United States

**Keywords:** virus dynamics, HIV-1, VRC01, mathematical modeling, ODE

## Abstract

VRC01 is a broadly neutralizing antibody that targets the CD4 binding site of HIV-1 gp120. Passive administration of VRC01 in humans has assessed the safety and the effect on plasma viremia of this monoclonal antibody (mAb) in a phase 1 clinical trial. After VRC01 infusion, the plasma viral load in most of the participants was reduced but had particular dynamics not observed during antiretroviral therapy. In this paper, we introduce different mathematical models to explain the observed dynamics and fit them to the plasma viral load data. Based on the fitting results we argue that a model containing reversible Ab binding to virions and clearance of virus-VRC01 complexes by a two-step process that includes (1) saturable capture followed by (2) internalization/degradation by phagocytes, best explains the data. This model predicts that VRC01 may enhance the clearance of Ab-virus complexes, explaining the initial viral decay observed immediately after antibody infusion in some participants. Because Ab-virus complexes are assumed to be unable to infect cells, i.e., contain neutralized virus, the model predicts a longer-term viral decay consistent with that observed in the VRC01 treated participants. By assuming a homogeneous viral population sensitive to VRC01, the model provides good fits to all of the participant data. However, the fits are improved by assuming that there were two populations of virus, one more susceptible to antibody-mediated neutralization than the other.

## Introduction

Passive administration of broadly neutralizing antibodies (bnAbs) in infected humanized-mice, macaques and humans has suggested that bnAb infusion may be a therapeutic modality against HIV-1 infection (1–3). One of the more potent bnAbs that has been isolated and characterized is VRC01 (4–6). VRC01 is a monoclonal antibody that recognizes the CD4 binding site of HIV gp120, emulating the binding of the CD4 receptor (5, 7).

**Figure 1:**
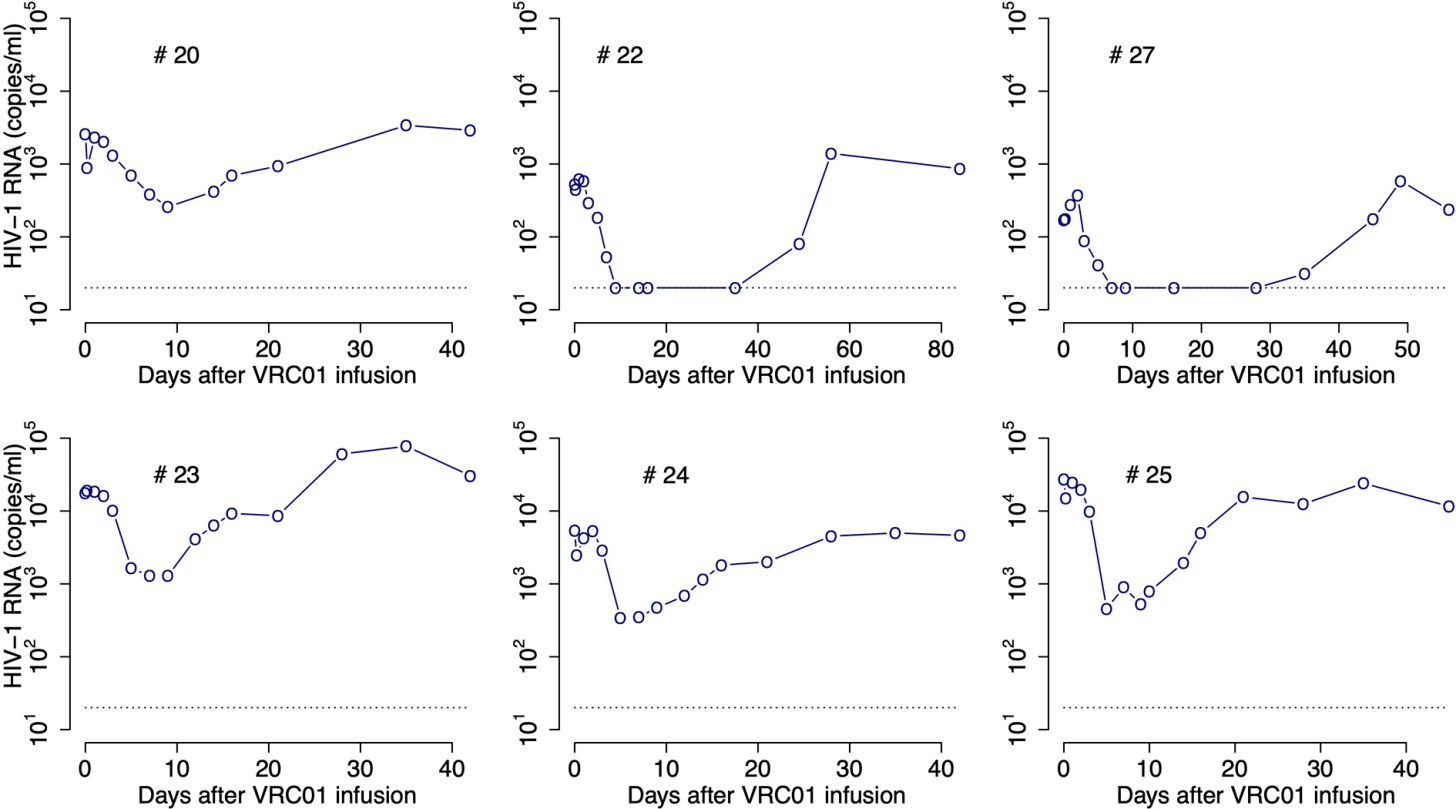
HIV-RNA levels in plasma after infusion of VRC01. Patient identifiers are given at the top of each graph.

To determine the pharmacokinetics, safety and effect of VRC01 on plasma viral load, this antibody was infused into HIV-1 chronically infected individuals in a phase 1 clinical trial (1, 8). After a single infusion of 40 mg/kg of VRC01, the plasma viral load was reduced by more than 1-log in 6/8 infected individuals, but there was no significant response in the other two participants (1). In the responding individuals, the major viral reduction occurred after a plateau phase that lasted about 2 days, which is longer than what is normally seen in infected participants under antiretroviral treatment (9, 10). In three participants, there was a rapid decay of virus immediately after VRC01 infusion, followed by a rebound to baseline over the next 24-48 hours. The other three responding participants presented a steady or an initial increase in the viral load to values higher than baseline. Both patterns were then followed by a decline in viral load that persisted but slowly returned to baseline as the VRC01 concentration declined (see **Figure 1**).

The aim of this paper is to obtain insight into the mechanisms that lead to these viral load dynamics. A pioneering study modeling the impact of antibodies during acute HIV infection adapted the basic model of virus dynamics to account for the possible effect of antibodies on viral infectivity, virion clearance, and infected cell death (11). More elaborated models including the explicit binding and dissociation of antibody to virus, in one or multiple steps have also been developed (12–14). More recently a mathematical model was used to determine if the bnAb 3BNC117 leads to antibody-dependent cellular cytotoxicity (ADCC) *in-vivo* (15). Here we develop mathematical models to fit the plasma HIV RNA data obtained after VRC01 infusion, with the goal of quantifying the mechanisms by which this mAb reduces viral load.

## Models and Results

### VRC01 Pharmacokinetics

After infusion of 40 mg/kg of VRC01, the serum antibody concentration decayed in a biphasic manner, similar to decays previously observed with other monoclonal antibodies (8, 16). The biphasic decay results from antibody distribution from the blood into the tissue followed by elimination from the body. As done previously (16, 17), we modeled these dynamics by using a two-compartment pharmacokinetic model presented in equation (1), where *A*_1_ and *A*_2_ represent the concentration of VRC01 in compartments one and two, respectively. In this model, VRC01 is infused at rate *R* for the period 0 < *t* < *T_end_* into the first compartment with volume *Vol*_1_. VRC01 is transported to the second compartment of volume *Vol*_2_ at rate *k*_12_, where it is cleared at rate *k*_0_. VRC01 is transported back to the first compartment at a rate *k*_21_. Following these assumptions, the model has the form,

(1) $$\begin{array}{l} \frac{dA_{1}}{dt}=R-k_{12}A_{1}+k_{21}A_{2}\frac{Vol_{2}}{Vol_{1}}\\ \frac{dA_{2}}{dt}=k_{12}A_{1}\frac{Vol_{1}}{Vol_{2}}-k_{21}A_{2}-k_{0}A_{2}\\ R=\{\begin{array}{c} \frac{A_{\max}}{T_{end}},t\leq T_{end}\\ 0,\;\;\;\;\;\;t>T_{end} \end{array} \end{array}.$$

We assume *T_end_* = 1 hour and *A_max_* equals the maximum measured VRC01 serum concentration. During the time of infusion (0 < *t* < *T_end_*) the equation for VRC01 concentration in the first compartment can be approximated by $\frac{dA_{1}}{dt}=R$, i.e., the antibody concentration *A*_1_ increases during the infusion at rate *R*, with solution

(2) $$A_{1}(t)=\frac{A_{\max}t}{T_{inf}},0<t<T_{end}$$

Substituting equation (2) into equation (1), we obtain for 0 < *t* < *T_end_*, $\frac{dA_{2}}{dt}=\frac{k_{12}A_{\max}tVol_{1}}{T_{end}Vol_{2}}-(k_{21}+k_{0})A_{2}.$ Since *A*_2_(0) = 0, the solution for *A*_2_(*t*) yields

(3) $$A_{2}(t)=\frac{k_{12}A_{\max}Vol_{1}}{T_{end}Vol_{2}}e^{-(k_{21}+k_{0})t}\int_{0}^{t}\tau e^{(k_{21}+k_{0})\tau }d\tau =\frac{k_{12}A_{\max}Vol_{1}}{T_{end}Vol_{2}(k_{21}+k_{0})}[(k_{21}+k_{0})t-1+e^{-(k_{21}+k_{0})t}].$$

Note that by substituting *A*_2_(*t*) back into Eq. (1) for *A*_1_ one can obtain a higher order approximation of *A*_1_(*t*). However, for our purposes with a short infusion the solution given by Eq. (2) suffices.

At the end of the infusion (i.e., *t* = *T_end_*) the predicted Ab concentration in the first and second compartments are *A*_1_(*T_end_*) = *A_max_*, and

$$A_{2}(T_{end})=\frac{k_{12}A_{\max}Vol_{1}}{Vol_{2}(k_{21}+k_{0})}[\frac{1}{(k_{21}+k_{0})T_{end}}\{e^{-(k_{21}+k_{0})t}-1\}+1.]$$

After the end of infusion, *t*>*T_end_*, *R*=0, and the antibody concentration decays as,

(4) $$A_{1}(t)=A_{\max}[ke^{-\lambda_{1}(t-T_{end})}+(1-k)e^{-\lambda_{2}(t-T_{end})}],t>T_{end}.$$

The parameters *λ*_1_ and *λ*_2_ are the eigenvalues of the system in equation (1) when *R* = 0, with form $\lambda_{1,2}=\frac{1}{2}(k_{21}+k_{0}+k_{12})\pm \sqrt{{\left(k_{21}+k_{0}-k_{12}\right)}^{2}+4k_{21}k_{12}}.$ The parameter *k* is obtained by equating the derivatives of *A*_1_(*t*) from equations (1) and (4) when *t* = *T_end_*, yielding $A_{\max}(-\lambda_{1}k-\lambda_{2}(1-k))=-k_{12}A_{\max}+\frac{k_{21}A_{2}(T_{end})Vol_{2}}{Vol_{1}}$ or $k=\frac{k_{12}A_{2}(T_{end})Vol_{2}}{Vol_{1}A_{\max}(\lambda_{2}-\lambda_{1})}+\frac{\lambda_{2}-k_{12}}{\lambda_{2}-\lambda_{1}},$ which upon substituting *A*_2_(*T_end_*) gives,

(5) $$k=\frac{k_{21}k_{12}}{\left(\lambda_{2}-\lambda_{1})(k_{21}+k_{0}\right)}\left[\frac{1}{(k_{21}+k_{10})T_{end}}\{e^{-(k_{21}+k_{0})t}-1\}+1\right]+\frac{\lambda_{2}-k_{12}}{\lambda_{2}-\lambda_{1}}.$$

Notice that the values of *λ*_1_ *, λ*_2_ and *k* do not depend on the values of *Vol*_1_ and *Vol*_2_. Therefore, the behavior of *A*_1_(*t*) in equations (2) and (4) does not depend on *Vol*_1_ and *Vol*_2_. However, as we show below, *A*_2_(*t*) does depend on the volume ratio, $\frac{Vol_{1}}{Vol_{2}}.$

**Figure 2:**
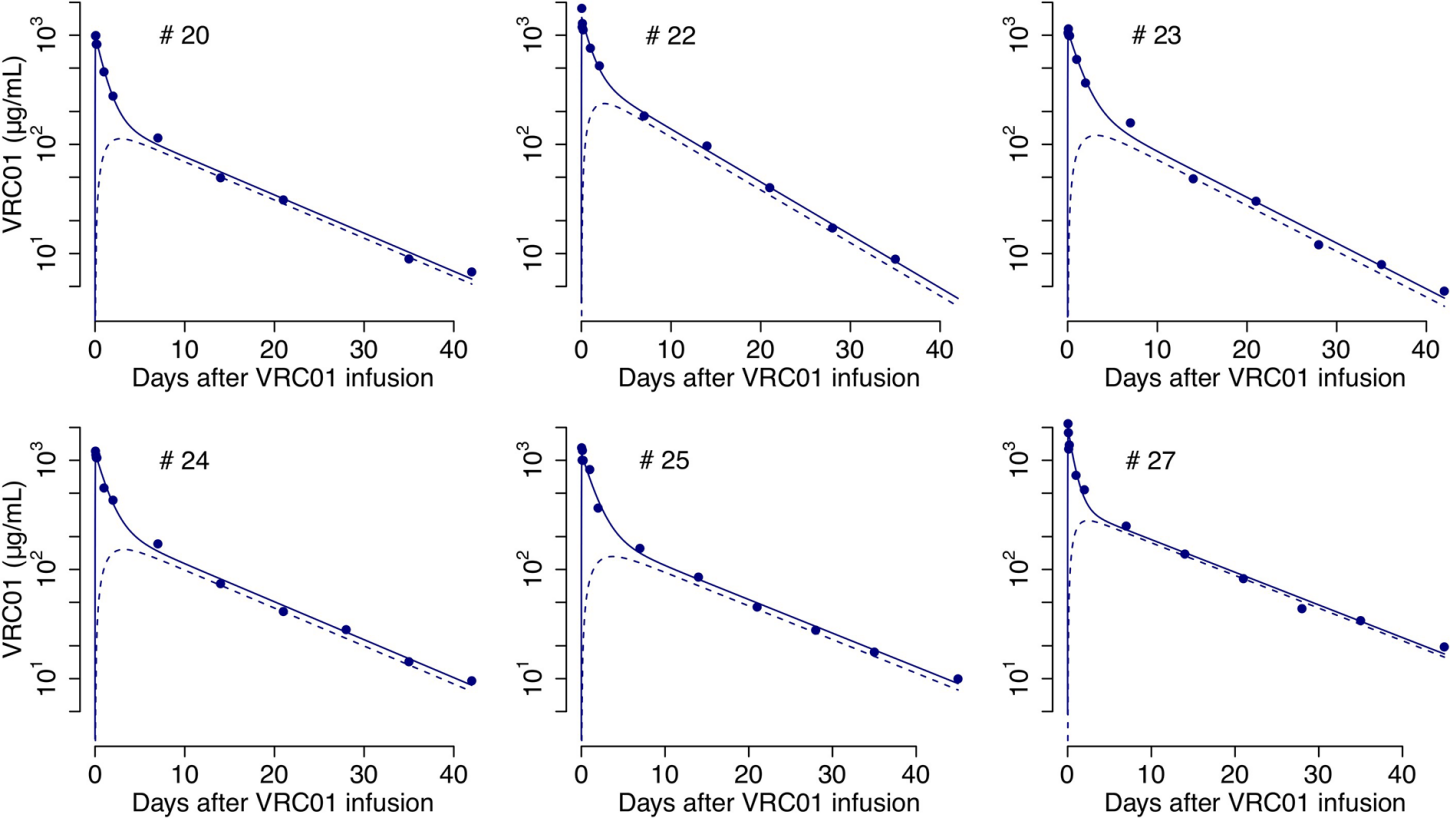
Best-fits of the two-compartment model in equations (2)-(6) to the PK data from infected individuals. Blue circles represent the measured VRC01 serum concentration, and solid lines the best fit from the model in equations (2) and (4) of the VRC01 concentration in the blood. Dashed lines represent the predicted concentration of VRC01 in the second compartment from the model in equations (3) and (6), assuming plasma volume, *Vol*_1_ = 3*L* and $Vol_{2}=\frac{k_{12}Vol_{1}}{k_{21}}$ (see **Table S1** for each participant’s parameter estimates).

We fit *A*_1_(*t*) given by equations (2) and (4) to the VRC01 serum concentration from the six infected individuals that responded to the mAb, estimating the rates of VRC01 distribution from blood to tissues, clearance, and transport from tissue to plasma: *k*_12_, *k*_0_ and *k*_21_, respectively. From the best fits (**Figure 2**) we obtain estimates for the clearance rate, *k*_0_, the rate of distribution from blood to tissues, *k*_12_, and rate of distribution from tissues to blood, *k*_21_, to be 0.09 day^-1^, 0.13 day^-1^ and 0.7 day^-1^, respectively (see the estimate for each participant in **Table S1**).

The VRC01 concentration was also measured in a group of uninfected, *i.e.* aviremic, volunteers in whom the same amount of VRC01 was infused. Doing the same analysis, we found that the biphasic decline was not significantly different between infected and aviremic participants, suggesting that the presence of HIV in infected participants did not significantly perturb their plasma VRC01 concentrations. For that reason, for the viral kinetic models in the following sections we simply assumed that the VRC01 concentration that affects the measured serum viremia, *A*(*t*), can be determined by the PK model, *i.e.*, *A*(*t*) = *A*_1_(*t*).

In more complex models where one also models the HIV-1 levels in tissue one could use *A*_2_(*t*) for the interactions of HIV-1 with VRC01 in tissue. We obtained a closed form solution for *A*_2_(*t*) when *t* > *T_end_*, by plugging in the solution of *A*_1_(*t*) in equation (4) into the differential equation for *A*_2_(*t*), in equation (1), yielding,

(6) $$\begin{array}{l} A_{2}(t)=A_{2}(T_{end})e^{-(k_{21}+k_{0})(t-T_{end})}+\frac{k_{12}A_{\max}Vol_{1}}{Vol_{2}}e^{-(k_{21}+k_{0})t}\left[k\int_{T_{end}}^{t}e^{(k_{21}+k_{0}-\lambda_{1})\tau }d\tau +(1-k)\int_{T_{end}}^{t}e^{(k_{21}+k_{0}-\lambda_{2})\tau }d\tau \right]\\ =A_{2}(T_{end})e^{-(k_{21}+k_{0})(t-T_{end})}+\frac{k_{12}A_{\max}Vol_{1}}{Vol_{2}}e^{-(k_{21}+k_{0})t}\{\left[\frac{k}{k_{21}+k_{0}-\lambda_{1}}+\frac{\left(1-k\right)}{k_{21}+k_{0}-\lambda_{2}}\right]e^{-(k_{21}+k_{0})(t-T_{end})}+\\ \left[\frac{\lambda_{1}ke^{-\lambda_{1}(t-T_{end})}}{k_{21}+k_{0}-\lambda_{1}}-\frac{\lambda_{2}(1-k)e^{-\lambda_{2}(t-T_{end})}}{k_{21}+k_{0}-\lambda_{2}}\right]\}. \end{array}$$

Unlike *A*_1_(*t*), the dynamics of *A*_2_(*t*), as presented in equations (3) and (6), depends on the ratio$\frac{Vol_{1}}{Vol_{2}}.$ Without knowledge of how VRC01 is distributed in tissues, one cannot determine *A*_2_(*t*). However, if we assume that the transport of the Ab keeps a balanced total flow imposing the constraint, *k*_21_ *Vol*_2_ = *k*_12_ *Vol*_1_ (17), then, assuming that *Vol*_1_ = 3*L* corresponds to the plasma volume one can estimate *Vol*_2_ and predict *A*_2_(*t*). Predictions of the VRC01 concentration in compartment two under this assumption, using the parameter estimates from fitting *A*_1_(*t*) to the serum VRC01 concentration samples given in **Table S1**, are presented in **Figure 2**.

### Virus Dynamics Modeling Approach

#### Description of the Virus Load Data in the Presence of VRC01

After a single passive administration of 40 mg/kg of VRC01, the change in plasma viral load in the responding infected individuals (with ID #20, #22, #23, #24, #25, and #27) can be separated into three phases (1): (i) an initial period during which there is a rapid viral decline followed by a rebound to baseline (in participants #20, #24, and #25), a plateau phase (in participant #23), or an initial viral increase in viral load (in participants #22 and #27). This initial period is followed by (ii) a long-term viral decline of about 1-log, and finally (iii) a viral rebound as the antibody concentration declines (see **Figure 1**).

The initial period lasted about 2-3 days. Using the viral load measurements at baseline and 4 hours after VRC01 infusion, we estimated the viral load reduction in the three participants that presented a rapid decline and rebound, and found viral decay slopes of 5, 4 and 3 day^-1^ in participants #20, #24 and #25, respectively. We further estimated the slope of the long-term decay beginning after the initial viral load rebound for the four participants whose viral load remained over the limit of detection (#20, #23, #24 and #25). We found that the long-term decline had slopes ranging from 0.28 to 0.78 day^-1^. These decline slopes are smaller than those estimated during potent ART of about 1 day^-1^ (9, 10, 18). Finally, between 5 or 10 days after VRC01 infusion, the viral load began to rebound to baseline values.

#### Modeling Approach

To model the virus dynamics in participants receiving VRC01, we modified the basic model of virus dynamics (19, 20). The basic model includes only the key players during HIV infection, but it has been able to describe the decline of the viral load in chronically infected participants during the first couple of weeks after receiving antiretroviral therapy (ART), and also the viral rebound after ART cessation (10, 21, 22). This basic model includes target cells for HIV, *T*, productively infected cells, *I*, and free virus, *V*. In the model target cells are created (e.g. in the thymus) at a constant rate *λ* and have a net per capita loss rate *d*. Target cells are infected by the virus and become productively infected with rate constant *β*. Productively infected cells, *I*, die at per capita rate *δ* and produce virus at a rate *p* per cell. Finally, free virus is cleared at rate *c* per virion. Under these assumptions the basic model has the form,

(7) $$\begin{array}{c} \frac{dT}{dt}=\lambda -dT-\beta VT\\ \frac{dI}{dt}=\beta VT-\delta I\\ \frac{dV}{dt}=pI-cV \end{array}.$$

Due to abortive HIV infection (23, 24), only a small fraction, *f*, of cells that are infected become productively infected (1 - *f* would be the fraction abortively infected). We included this feature by modifying the infection term to *fβVT* in the infected cell equation. Furthermore, it has been suggested that the death rate of infected cells is not constant, as in equation (7), but it might vary proportionally to the density of effector cells (i.e. δ∝E(t), being *E*(*t*) the effector cell density) (25–27). Holte et al. (28) presented one of the simplest versions of this approach where the effector cell density in turn depends on the infected cell density with form $E(t)\propto I{\left(t\right)}^{\omega -1}$. In this case, the death rate of infected cells is expressed as $\delta =\hat{\delta }I{\left(t\right)}^{\omega -1}$ (notice that assuming *ω* = 1 yields a constant death rate of infected cells as in equation (7)). Adding these features, we have a virus dynamic model of the form,

(8) $$\begin{array}{c} \frac{dT}{dt}=\lambda -dT-\beta VT\\ \frac{dI}{dt}=f\beta VT-\hat{\delta }I^{\omega }\\ \frac{dV}{dt}=pI-cV \end{array}.$$

#### Modeling the Effect of VRC01 on HIV-1 Viral Load

As presented in (11), the simplest way to model the effect of antibodies on viral infection is by increasing or decreasing parameters in the basic model corresponding to processes that HIV-specific antibodies might affect. For example, neutralization of the virus due to opsonization can be included in this basic model, by reducing the infection rate constant *β* in equation (8) by a factor 1 + *αA*(*t*), where *α* is a constant (11). Therefore, in the presence of HIV-specific antibodies target cells would become infected at rate $\frac{\beta VT}{1+\alpha A(t)}.$

One can deduce this simplification by including into the basic model reversible binding of the antibody to the virus to produce neutralized immune complexes, *C*. We assume antibodies bind to the virus with rate constant *k_on_* and dissociates from it with rate constant *k_off_*. Assuming that immune complexes are cleared from the plasma at rate *γ*, one ends up with a model of the form,

(9) $$\begin{array}{c} \frac{dT}{dt}=\lambda -dT-\beta VT\\ \frac{dI}{dt}=f\beta VT-\hat{\delta }I^{\omega }\\ \frac{dV}{dt}=pI-cV-k_{on}VA(t)+k_{off}C\\ \frac{dC}{dt}=k_{on}VA(t)+k_{off}C-\gamma C \end{array}.$$

Assuming that immune complexes come into a quasi-steady state with the viral load, one obtains: *k_on_VA*(*t*) = (*k_off_* + *γ*)*C*. Thus, the fraction of free/non-neutralized virus in the presence of HIV-specific antibodies, $\frac{V}{V+C},$ will be equal to $\frac{1}{1+\frac{k_{on}A(t)}{k_{off}+\gamma }}$. Defining $\alpha =\frac{k_{on}}{k_{off}+\gamma },$ the model in equation (9) can be simplified to the form,

$$\begin{array}{c} \frac{dT}{dt}=\lambda -dT-\frac{\beta V_{T}T}{1+\alpha A(t)}\\ \frac{dI}{dt}=\frac{f\beta V_{T}T}{1+\alpha A(t)}-\hat{\delta }I^{\omega } \end{array},$$

where *V_T_* = *V* + *C* is the total amount of virus per unit volume and the $\frac{dV}{dt}$ and $\frac{dC}{dt}$ equations are the same as in Eq. (9). Assuming that immune complexes are cleared at the same rate as free virus (*i.e.*, *γ* = *c*), as would be the case *in vitro* where *γ* = *c* = 0, and then adding the equations for $\frac{dV}{dt}$ and $\frac{dC}{dt}$ one finds

(10) $$\begin{array}{c} \frac{dT}{dt}=\lambda -dT-\frac{\beta V_{T}T}{1+\alpha A(t)}\\ \frac{dI}{dt}=\frac{f\beta V_{T}T}{1+\alpha A(t)}-\hat{\delta }I^{\omega }\\ \frac{dV_{T}}{dt}=pI-cV_{T} \end{array}.$$

Note that the model in equation (10) has the same structure of the basic model in equation (8), with the infectivity reduction proposed in Tomaras et al. (11). Also notice that from this approach, one may glean information about the dissociation constant $K_{d}=\frac{k_{off}}{k_{on}}$ from the parameter *α*. For simplicity from now on variable *V* will represent total viral load (i.e., *V_T_*) unless a $\frac{dC}{dt}$ equation is distinctly specified for virus-VRC01 complexes. In the latter case *V* will represent free virus only.

To analyze the effect of virus neutralization by VRC01 on the viral load, we propose in the following sections adaptations of the models in equations (9) and (10), and show the best-fits of those adaptations to the HIV-RNA data. Model symbols and parameter values are described in **Table 1**.

**Table 1:** Description of model parameters.

Parameters	Description	Value	Unit	Reference
***f***	Fraction of target cells that after infection become productively infected cells.	0.05		(23, 24)
$\tilde{\delta }\hat{I}{\left(0\right)}^{\omega -1}$ ****	Initial productively infected cell decay rate.	1.5 (See *Materials and Methods* section)	day^-1^	(29)
***ω***	Parameter that quantifies the density dependent rate of infected cells.	Fitted		(28)
***c***	Clearance rate of virus in plasma.	23	day^-1^	(30)
***β_s_***, ***β_r_***	Density dependent infection rate for sensitive and less-sensitive virus.	See *Materials and Methods* section	ml day^-1^	
***λ***	Target cell production rate.	See *Materials and Methods* section	ml^-1^ day^-1^	
***d***	Death rate of target cells and cells that are not productively infected.	0.01	day^-1^	(31)
***p***	Virus production rate per infected cell	Fitted	day^-1^	
***α_s_***, ***α_r_***	Sensitive and less-sensitive virus neutralization sensitivity to VRC01 (delayed neutralization models)	Fitted	ml	
***τ***	Delay in the neutralization of VRC01 (delayed neutralization models)	Fitted	days	
$k_{off_{s}},k_{off_{r}}$	VRC01-virus neutralized complex dissociation rate (phagocytosis-based clearance models).	2.75	day^-1^	(32, 33)
$k_{on_{s}},k_{on_{r}}$	VRC01-senstive and less-sensitive virus neutralized complex formation rate (phagocytosis-based clearance models).	Fitted	ml day^-1^	
***γ***	Maximum clearance rate of immune complexes (phagocytosis-based clearance models).	Fitted	day^-1^	
***m***	Degradation rate of immune complexes captured by phagocytes (phagocytosis-based clearance models).	Fitted	day^-1^	
***K***	Carrying capacity of captured immune complexes (phagocytosis-based clearance models).	Fitted	copies ml^-1^	

### Delayed-Neutralization Model

During passive infusion of a potent broadly neutralizing antibody, such as VRC01, one would assume that infection of target cells would be significantly reduced when the serum concentration of the antibody is high. Thus, we would expect that the VRC01-mediated neutralization of the virus will block *de novo* infection events, and the rate of viral load decline will reflect the death rate of infected cells, similar to what is observed after initiation of therapy with protease, reverse transcriptase, and integrase strand transfer inhibitors (PIs, RTIS and InSTI, respectively) (10, 21, 22). However, the data shows that there is a delay, which is longer than the one observed after initiation of therapy with PIs, RTIs and InSTIs, before there is a major reduction of plasma viral load (10, 21, 22).

An empirical way to account for this delay, without explicitly explaining the mechanism behind it, is to assume that the presence of VRC01 decreases virus infectivity, as presented in the previous section, with a delay *τ* after the mAb infusion. Thus, in the presence of VRC01 target cells become infected at a rate $\hat{\beta }VT,$ where

(11) $$\hat{\beta }=\{\begin{array}{c} \overset{}{\beta }\\ \frac{\beta }{\left(1+\alpha A(t)\right)}, \end{array}\begin{array}{c} t<\tau \\ t\geq \tau \end{array}.$$

**Figure 3:**
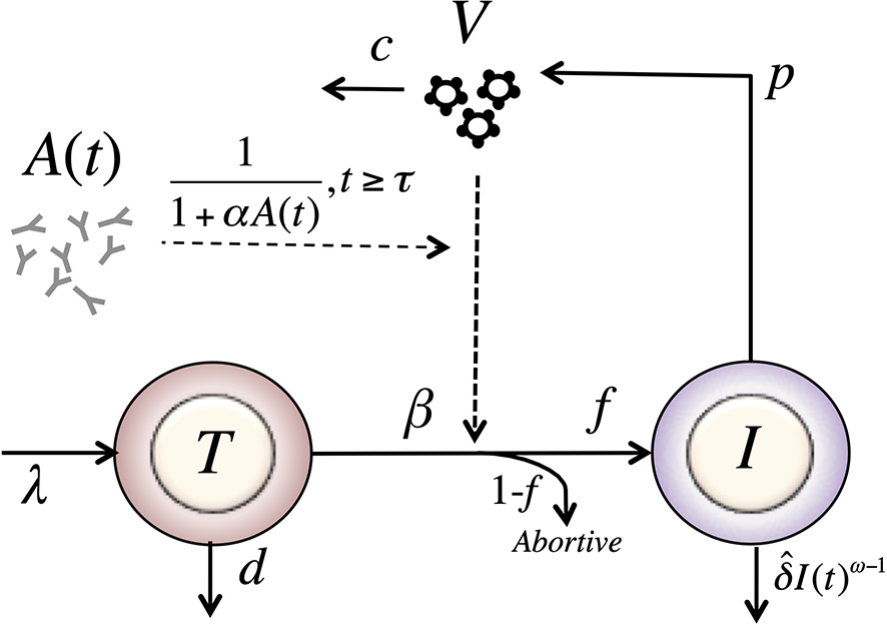
Schematic of the delayed-neutralization model. The model includes three variables: target cells (*T*), productively infected cells (*I*), and free virus (*V*), Parameters *λ*, *d* and *β* are the production, death and infection rates of target cells; *δ* is the death rate of infected cells; *p* and *c* are the production and clearance rates of free virus. Antibodies decrease the infectivity after a delay *τ*, with sensitivity *α*. The antibody concentration, *A*(*t*), is determined from the best fits of the two-compartmental model to VRC01 concnetration measurements for each individual (see text).

Adding this feature to the model in equation (8), we have a model with form (see **Figure 3**),

(12) $$\begin{array}{c} \frac{dT}{dt}=\lambda -dT-\hat{\beta }VT\\ \frac{dI}{dt}=f\hat{\beta }VT-\hat{\delta }I^{\omega }\\ \frac{dV}{dt}=pI-cV \end{array}.$$

**Figure 4:**
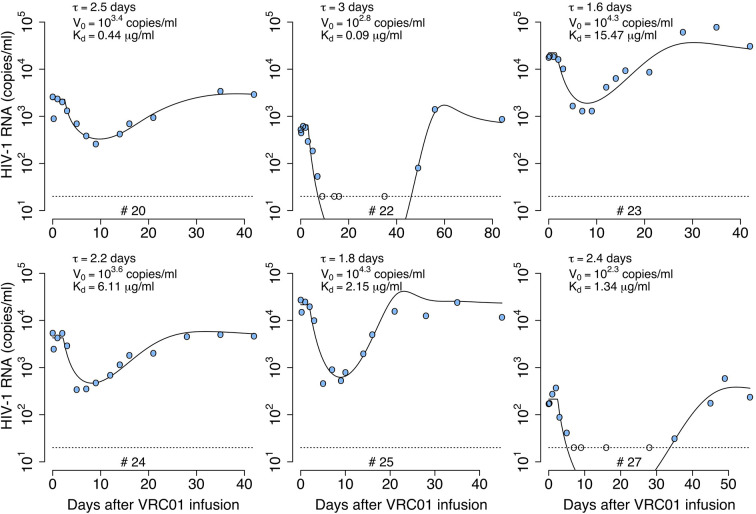
Best-fits of the delayed neutralization model, equation (12), to viral load data from participants receiving a single infusion of VRC01. Blue-filled and unfilled circles are the HIV-RNA over and below the limit of detection, respectively. Solid black lines are the best-fit of the model in equation (12) to the data. Fixed parameter values are described in **Table 1**. See all parameters estimates in **Table S2**. Other parameters and initial values were derived by assuming the system was in steady state before VRC01 infusion.

We fit the model in equation (12) to the viral load data after VRC01 infusion as described in the *Materials and Methods* section, estimating the parameters *τ*, *α*, *pT*(0), *V*(0) and *ω* (see individual parameter estimates in **Table S2**). From the best fits, as shown in **Figure 4**, we found that, in general, the model is able to predict a delay, followed by viral decay and rebound as observed in the viral load data after VRC01 infusion in all participants. However, the data for participants #24 and #25 appear to have a faster decline than that predicted by the model. In addition, the model predicts a median delay of *τ* = 2.3 days before the neutralization effect of VRC01 is observed in the viral load data. Using the relation $\alpha =\frac{k_{on}}{k_{off}+\gamma }$ from the previous section, assuming that *γ* = *c* = 23 day^-1^, and that *k_off_* is equivalent to the VRC01 off-rate constant measured *in vitro* on a YU2 gp120 subunit, i.e. *k_off_* = 2.75 day^-1^ (32, 33), with our estimate of *α* we calculate a dissociation coefficient $K_{d}=\frac{k_{off}}{k_{on}}$ of about 1.7 μg ml^-1^. This dissociation coefficient is higher than the value estimated *in vitro* [~0.3 μg ml^-1^ (32, 33)]. Nevertheless, the estimated dissociation coefficient seems reasonable, as viral rebound is observed when the VRC01 serum concentration has levels around 10 μg ml^-1^. Besides, the conditions in which the *in vitro K_d_* values are obtained may be significantly different than the *in vivo* conditions in chronically infected individuals, which may explain the difference in the estimates.

#### Delayed-Neutralization Model With Two Viral Populations

Lynch et al. (1) detected virus populations less sensitive to VRC01 a month after antibody infusion. It is possible that selection pressure leads to the growth of less sensitive (i.e. partially VRC01 resistant) populations when the antibody concentration is high (1). Here, we combine all the less sensitive virus into a second viral population with a different infectivity and sensitivity to VRC01, *β_r_* and *α_r_*, respectively. In the model, the less-sensitive virus population infects target cells at rate *β_r_V_r_T.* As in the one viral population model, the infectivity is only affected by VRC01 after a delay *τ*,

(13) $${\hat{\beta }}_{r}=\{\begin{array}{c} \beta_{r},\\ \frac{\beta_{r}}{\left(1+\alpha_{r}A(t)\right)}, \end{array}\begin{array}{c} t<\tau \\ t\geq \tau \end{array}$$

We include two more equations for the population of productively infected cells, *I_r_*, infected by the less-sensitive virus population, and the virus population *V_r_*, using the same structure as for the sensitive-population. Therefore, assuming *V_s_* represents the virus sensitive to VRC01, with infectivity and sensitivity to VRC01, *β_s_* and *α_s_*, respectively, the model in equation (12) is adjusted to

(14) $$\begin{array}{c} \frac{dT}{dt}=\lambda -dT-{\hat{\beta }}_{s}V_{s}T-{\hat{\beta }}_{r}V_{r}T\\ \frac{dI_{s}}{dt}=f{\hat{\beta }}_{s}V_{s}T-\hat{\delta }I_{s}^{\omega }\\ \frac{dI_{r}}{dt}=f{\hat{\beta }}_{r}V_{r}T-\hat{\delta }I_{r}^{\omega }\\ \frac{dV_{s}}{dt}=pI_{s}-cV_{s}\\ \frac{dV_{r}}{dt}=pI_{r}-cV_{r} \end{array}$$

**Figure 5:**
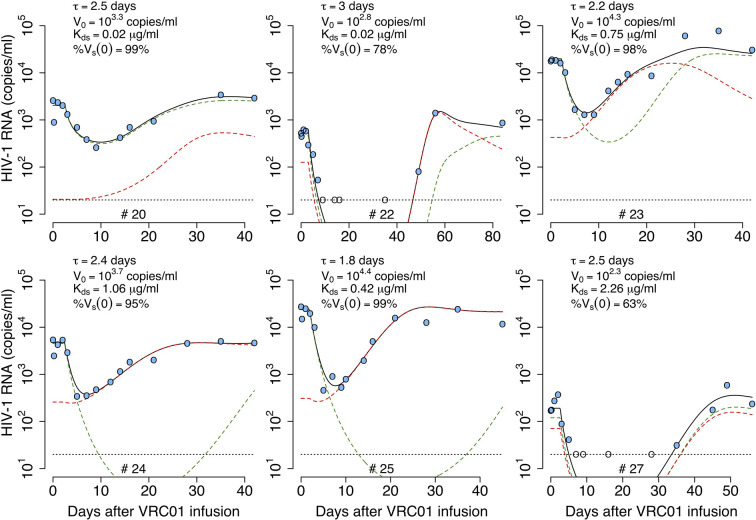
Best-fits of the two viral population delayed neutralization model, equation (14), to viral load data from participants receiving a single infusion of VRC01. Blue-filled and unfilled circles are the HIV-RNA over and below the limit of detection, respectively. Solid black lines are the best-fit of the model to the data (*V_s_* + *V_r_*). Green and red dashed lines show the sensitive (*V_s_*) and less sensitive (*V_r_*) virus concentration prediction of the model, respectively. Fixed parameter values are described in **Table 1**. See all parameter estimates in **Table S3**. Other parameters and initial values were derived by assuming the system was in steady state before VRC01 infusion.

As before, we fit the model in equation (14) to the viral load data, now estimating the parameters *τ*, *α_s_*, *α_r_*, % *V_s_*(0), *pT*(0), *V*(0) and *ω* (see best-fit parameter estimates in **Table S3**). As shown in **Figure 5**, this model also recapitulates the viral load data, but the fits to all participants data had similar or worst statistical support compared to the delayed neutralization model with one viral population (See **Table S3**). Nevertheless, this model predicts a similar delay of *τ* = 2.4 days for the virus neutralization effect. The model also predicts 96% of the virus is sensitive to VRC01 at baseline. However, because the less-sensitive virus population is less efficiently neutralized it can ultimately dominate the viral population (e.g., participants #23, 24 and 25, **Figure 5**). Since, the less sensitive viral population is important in viral rebound, the model predicts a lower dissociation constant of VRC01 for the sensitive virus, *K_ds_*~0.6 μg ml^-1^, than the one predicted with a model with only one viral population of 1.7 μg ml^-1^. Interestingly, this *K_ds_* value is close to the average *K_d_* value of 0.3 μg ml^-1^ estimated *in-vitro*.

A disadvantage of this model is that it does not provide a mechanistic explanation of the initial delay in the neutralization effect. The delay in the viral load decline in chronically infected individuals initiating ART is thought to be due to a combination of the pharmacological delay after oral uptake of the drug and the step in the viral cycle at which the drug acts (9, 10, 34). However, in the case of VRC01, the mAb is infused directly into bloodstream, and it is not clear why there is such a long delay. One possibility is that the delay reflects the time for the infused mAb to be transported into tissues where the majority of virus replication occurs and accumulate to a high enough concentration to effectively neutralize the virus. Another possibility is that the delay may reflect other mAb-mediated mechanisms of action against the virus. We will explore the latter case in the following sections.

### Phagocytosis-Based Saturated Clearance Model

If one assumes that the viral load is measured accurately, then the rapid decay of virus immediately after VRC01 infusion followed by a rebound to baseline over the next 24-48 hours in participants #20, #24 and #25 needs to be explained. Further, in participant #27, there was an initial increase in the viral load to values higher than baseline. These observations may indicate that after VRC01 infusion, there is not a physiological delay, but rather that VRC01 has an immediate effect on the virus. The early fast decay seen in some of the individuals have slopes ≥3 day^-1^, faster than the viral decline rate seen after the initiation ART, of about 1 day^-1^, suggesting that this fast decline does not reflect the death of infected cells. Rather, VRC01 maybe disrupting the viral set point by enhancing the clearance of the virus. The simplest way to codify this effect in the basic model is by adding an antibody-dependent enhanced factor to the virus clearance rate (11):

(15) $$\frac{dV}{dt}=pI-c[1+\gamma A(t)]V$$

**Figure 6:**
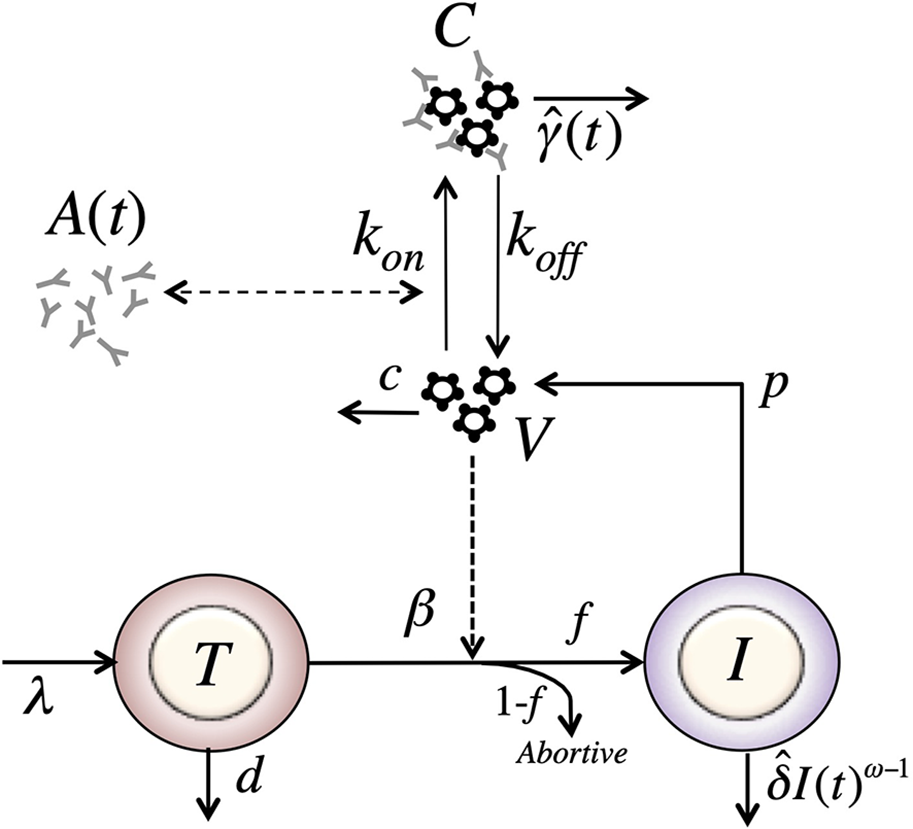
Schematic of the model with reversible binding of Ab to HIV and a time-varying clearance rate of immune complexes. The model include four variables: target cells (*T*), productively infected cells (*I*), free virus (*V*), and VRC01-HIV immune complexes (*C*). Parameters *λ*, *d* and *β_s_* are the production, death and infection rates of target cells; *δ* is the death rate of infected cells; *p* and *c* are the production and clearance rates of free virus; *k_on_* and *k_off_* are the binding and dissociation rate constants of VRC01 to free virus; $\hat{\gamma }(t)$ is the time varying clearance rate of VRC01-HIV immune complexes. The antibody concentration, *A*(*t*), is determined from the best-fit of the two-compartmental model to VRC01 concentration measurements for each individual (see text).

This approach would account for the rapid early decay of the virus but not for the fast rebound. To have an early viral decline and a rebound, the model has to include a viral clearance rate that varies over time. A simple model to account for this effect can be obtained by adjusting the model in equation (9) assuming a time varying clearance of immune complexes. Thus, the equation for immune complexes in equation (9) can be adjusted to (see **Figure 6**),

(16) $$\frac{dC}{dt}=k_{on}VA(t)-k_{off}C-\hat{\gamma }(t)C.$$

Assuming that the viral load reflects the total viral load, i.e. the free- and complexed-virus *V* + *C*, the value of $\hat{\gamma }(t)$ has to be initially greater than the clearance of free virus, *c*, to disrupt the set point leading to an initial fast decay. Then, at some point the value of $\hat{\gamma }(t)$ has to decrease below *c* to account for the viral rebound.

One plausible biological explanation for this behavior of $\hat{\gamma }(t)$ is that immune complexes, *C*, are cleared as they interact with Fc receptors on phagocytes with a phenomenological carrying capacity *K*. Then, when the concentration of immune complexes is low, *C* << *K*, $\hat{\gamma }(t)$ will be high. As VRC01 interacts with free virus, the concentration of immune complexes, *C*, increases, and the likelihood of interaction of immune complexes with phagocytes decreases as fewer free Fc receptors might be available, or other Fc-Fc receptor interaction obstacles may appear, reducing the clearance rate. We can describe this phenomenologically using a clearance rate with the form $\hat{\gamma }(t)=\gamma \frac{K}{K+C}.$ At low concentrations, *c*, immune complexes are cleared at a rate close to *γ.* If VRC01 enhances the clearance of virus by forming immune complexes, this would be reflected in the model by *γ* > *c*, and one would expect a rapid viral decay disrupting the steady state. As more complexes form, they might not be cleared as efficiently, if the phagocytic capacity of the host becomes exhausted (35–38), and when $\gamma \frac{K}{K+C}<c,$ we would expect a rebound in the viral load. Under these assumptions, the model has the form,

(17) $$\begin{array}{c} \frac{dT}{dt}=\lambda -dT-\beta VT\\ \frac{dI}{dt}=f\beta VT-\hat{\delta }I^{\omega }\\ \frac{dV}{dt}=pI-cV-k_{on}VA(t)+k_{off}C\\ \frac{dC}{dt}=k_{on}VA(t)+k_{off}C-\gamma \frac{K}{K+C}C \end{array}.$$

We fit the model in equation (17) to the data, estimating the parameters *γ*, *K, pT*(0), *k_on_* and *ω*. As before, we assume that *k_off_* is equivalent to the off-rate constant of VRC01 measured *in vitro* equal to 2.75 day^-1^ (32, 33). As presented in **Figure S1**, this model is not able to capture the early fast decay and rebound of the viral load after VRC01 infusion nor the long-term decline. Thus, this model does not improve the fits of the two previous models in any of the participants (See **Table 2**).

**Table 2:** BIC values from the best fits of each model to each participant’s viral load data.

ID	BIC					
	DNM		PSCM		PLCM	
	1 Viral pop.	2 Viral pops.	1 Viral pop.	2 Viral pops.	1 Viral pop.	2 Viral pops.
**#20**	-39.1	-35.7	-28.7	-39.4	**-54.0**	-49.7
**#22**	-4.0	1.1	-13.3	-38.0	**-50.9**	-45.3
**#23**	-34.8	-34.2	-30.4	-29.0	-34.8	**-42.1**
**#24**	-44.0	-45.8	-27.8	-27.6	-53.4	**-68.7**
**#25**	-32.2	-31.7	-18.7	-13.3	-26.0	**-34.6**
**#27**	-37.9	-32.0	-15.6	-25.7	**-40.4**	-36.1

DNM, Delayed neutralization model; PSCM, Phagocytosis-based saturated clearance model; PLCM, Phagocytosis-based logistic clearance model. In bold, the lowest BIC of each row.

#### Phagocytosis-Based Saturated Clearance Model With Two Viral Populations

As before, we considered a variant of the previous model including a preexisting viral population less sensitive to VRC01. VRC01 also binds to this second viral population to form VRC01-HIV complexes, *C_r_*, but with reduced affinity We assume in this model that phagocytic cells capture both types of immune complexes, *C_s_* and *C_r_*. If the same maximum clearance rate *γ* is used for both viral populations, the model makes a similar prediction than with the one with only one viral population (simulations not shown). Therefore, we assume that the immune complexes, *C_s_* and *C_r_*, have clearance rates *γ_s_* and *γ_r_*. In this case, the clearance rate of immune complexes has the form ${\hat{\gamma }}_{s}(t)=\gamma_{s}\frac{K}{K+\gamma_{s}C_{s}+\gamma_{r}C_{r}}$ and ${\hat{\gamma }}_{r}(t)=\gamma_{r}\frac{K}{K+\gamma_{s}C_{s}+\gamma_{r}C_{r}}$ for immune complexes with sensitive and resistant virus, respectively. Notice that the clearance of immune complexes depends on the competition of *C_s_* and *C_r_* to be captured by Fc receptors, and the advantage of one over the other depends on the rates *γ_s_* and *γ_r_*. Because VRC01 has higher affinity for sensitive virus, *V_s_*, than the partially resistant virus, *V_r_*, the *V_s_*-Ab complexes should have more antibody in them, and hence be taken up preferentially by phagocytes, i.e., we expect *γ_s_* > *γ_r_*. Therefore, the sensitive virus will decay faster in the initial hours after VRC01 infusion, but the clearance of the less-sensitive virus might be reduced leading to an early increase of this population, reflected in the early viral rebound. With these assumptions, the model has the form,

(18) $$\begin{array}{c} \frac{dT}{dt}=\lambda -dT-\beta_{s}V_{s}T-\beta_{r}V_{r}T\\ \frac{dI_{s}}{dt}=f\beta_{s}V_{s}T-\hat{\delta }I_{s}^{\omega }\\ \frac{dI_{r}}{dt}=f\beta_{r}V_{r}T-\hat{\delta }I_{r}^{\omega }\\ \frac{dV_{s}}{dt}=pI_{s}-cV_{s}-k_{on_{s}}V_{s}A(t)+k_{off}C_{s}\\ \frac{dV_{r}}{dt}=pI_{r}-cV_{r}-k_{on_{r}}V_{r}A(t)+k_{off}C_{r}\\ \frac{dC_{s}}{dt}=k_{on_{s}}V_{s}A(t)-k_{off}C_{s}-{\hat{\gamma }}_{s}(t)-\gamma_{s}\frac{K}{K+\gamma_{s}C_{s}+\gamma_{r}C_{r}}C_{s}\\ \frac{dC_{r}}{dt}=k_{on_{r}}V_{r}A(t)-k_{off}C_{r}-\gamma_{r}(t)-\gamma_{s}\frac{K}{K+\gamma_{s}C_{s}+\gamma_{r}C_{r}}C_{r} \end{array}.$$

**Figure 7:**
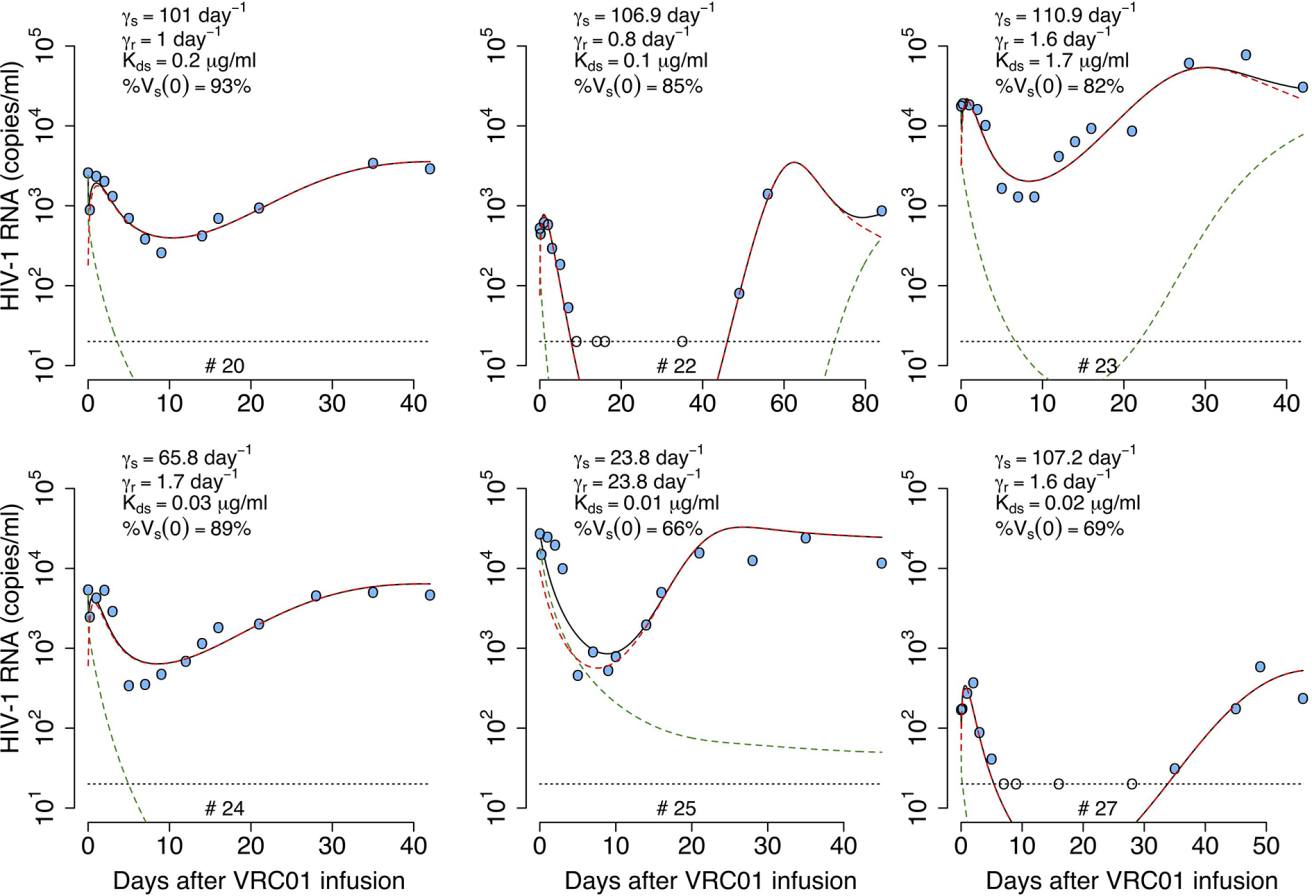
Best-fits of the phagocytosis-based saturated clearance model with two viral populations, equation (18), to viral load data from participants receiving a single infusion of VRC01. Blue-filled and unfilled circles are the HIV-RNA over and below the limit of detection, respectively. Solid black lines are the prediction of best fit of the model in equation (18) to the data (*V_s_* + *V_r_* + *C_s_* + *C_r_*). Green and red dashed lines show the sensitive (*V_s_* + *C_s_*) and less sensitive (*V_r_* + *C_r_*) virus concentration prediction of the model, respectively. Fixed parameter values are described in **Table 1**. See all parameter estimates in **Table S5**. Other parameters and initial values were derived by assuming the system was in steady state before VRC01 infusion.

We fit the model in equation (18) to the data, estimating the parameters *γ_s_*, *γ_r_*, *K, pT*(0),$k_{on_{s}}$, $k_{on_{r}}$, %*V_s_*(0) and *ω* (see best-fit parameter estimates in **Table S5**). In general, this model is able to reproduce the data well (**Figure 7**) and predicts a fast viral decline and rebound in most of the cases (except participant #25). The fast decline is due to the loss of sensitive virus to VRC01, and the rebound is due to the formation of VRC01/less-sensitive virus complexes. However, this model only improved the fits to the data from participants #20 and #22 compared to all previous models. Nonetheless, from the fits the model predicts a dissociation coefficient for the sensitive virus, $\left(K_{d_{s}}=\frac{k_{off}}{k_{on_{s}}}\right)$ around 0.04 μg ml^-1^, smaller than the *K_d_* estimates of VRC01 *in-vitro*. However, because this model predicts that most of the virus during the early rebound comes from the less-sensitive population, the value of $K_{d_{s}}$ might be overestimated. The model also predicts that the sensitive virus corresponds to the majority (~84%) of the initial viral population. The model also predicts that the ratio between the capture rates of the immune complexes, $\frac{\gamma_{s}}{\gamma_{r}},$ is between 10 and 10^2^ (except participant #25), implying that complexes *C_s_* are much more likely to be cleared than complexes *C_r_*.

In summary, the model predicts that during the first hours after VRC01 infusion, sensitive immune complexes are formed and are cleared faster than free virus by phagocytic activity (green dashed lines in **Figure 7**), explaining the initial fast decay. As the phagocytic capture is smaller for less sensitive immune complexes, fewer such complexes (red dashed lines in **Figure 7**) are cleared producing a fast rebound over the next hours. Neutralization of virus by VRC01 leads to a reduction of *de novo* infection events and coupled with death of already infected cells leads to a decrease of productively infected cells reflected in the viral decay observed over the next couple of days. While the concentration of VRC01 is still high enough to affect the sensitive virus, the less VRC01-sensitive virus population might be selected (red dashed lines in **Figure 7**) producing the final rebound in viral load.

While the model accurately captures the early viral decline, and rebound in participant #20, it does not do so for participants #24 and #25. Thus, we consider another variant of the model.

### Phagocytosis-Based Logistic Clearance Model

**Figure 8:**
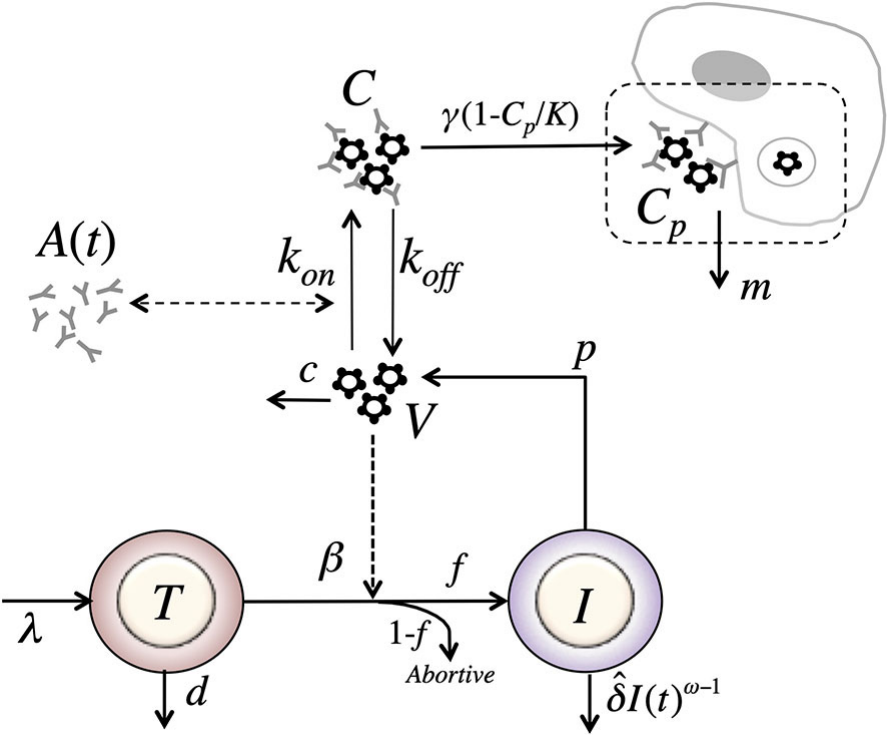
Schematic of the phagocytosis-based logistic clearance model (for one virus population) used to analyze the virus dynamics after VRC01 infusion. The model includes five variables: target cells (*T*), productively infected cells (*I*), free virus (*V*), VRC01-HIV immune complexes (*C*), and complexes cleared by phagocytes (*C*). Parameters *λ*, *d* and *β_s_* are the production, death and infection rates of target cells; *δ* is the death rate of infected cells; *p* and *c* are the production and clearance rates of free virus; *k_on_* and *k_off_* are the binding and dissociation rate constants of VRC01 to free virus; *γ*, *K* and *m* are the phagocytosis mediated maximum clearance rate, carrying capacity and degradation rate of VRC01-HIV immune complexes. The antibody concentration, *A*(*t*), is determined from the best-fits of the two-compartmental model to VRC01 concentration measurements for each individual (see text).

In our final model, we assume that immune complexes first become “captured” immune complexes, *C_p_*, i.e. bind to Fc receptors. However, in a second step the captured complexes need to be internalized and degraded. Modeling this two-step process assuming a logistic form for capture of complexes with carrying capacity *K*, and an internalization and degradation rate *m* of captured complexes, we obtain a model of the form (**Figure 8**)

(19) $$\begin{array}{c} \frac{dT}{dt}=\lambda -dT-\beta VT\\ \frac{dI}{dt}=f\beta VT-\hat{\delta }I^{\omega }\\ \frac{dV}{dt}=pI-cV-k_{on}VA(t)+k_{off}C\\ \frac{dC}{dt}=k_{on}VA(t)-k_{off}C-\gamma \left(1-\frac{C_{p}}{K}\right)C\\ \frac{dC_{p}}{dt}=\gamma \left(1-\frac{C_{p}}{K}\right)C-mC_{p} \end{array}.$$

Notice that if we assume that the captured complexes, *C_p_*, are in quasi-stationary state, then$\frac{dC_{p}}{dt}=0,$ and $>{\bar{C}}_{p}=\frac{\frac{\gamma }{m}KC}{K+\frac{\gamma }{m}C}.$ Substituting ${\bar{C}}_{p}$ in equation (19), we get, $\frac{dC}{dt}=k_{on}VA(t)-k_{off}C-\gamma \frac{K}{K+\frac{\gamma }{m}C}C,$ identical to the form of the equation for immune complexes in equation (17). Therefore, the model in equation (17) is a special case of the model in equation (19) where $\frac{\gamma }{m}=1$ (the same applies for the case with two viral populations).

Notice that as described in the previous model, if VRC01 enhances the clearance of virus by forming immune complexes, then *γ* > *c* and one would expect a rapid viral decay disrupting the steady state when the levels of *C_p_* are low. Then, as immune complexes are captured and degraded by phagocytes with a carrying capacity *K*, the early decline gets disrupted when the number of captured complexes *C_p_* approaches the carrying capacity. Thus, when $\gamma \left(1-\frac{C_{p}}{K}\right)<c$ or equivalently when $\left(\frac{C_{p}}{K}\right)>1-\frac{c}{\gamma }$ the model predicts a switch to a viral increase rather than a decease. This is easy to see since the rate of change of the total virus, $\frac{d(V+C)}{dt}=pI-cV-\gamma \left(1-\frac{C_{p}}{K}\right)C.$ When $\gamma \left(1-\frac{C_{p}}{K}\right)=c,\frac{d(V+C)}{dt}=pI-c(V+C)$ and because *c* is large the system will rapidly reach a quasi-steady state where total virus production and clearance balance. When $\gamma \left(1-\frac{C_{p}}{K}\right)<c,$ total viral clearance will be less than total production and the total amount of virus will increase.

**Figure 9:**
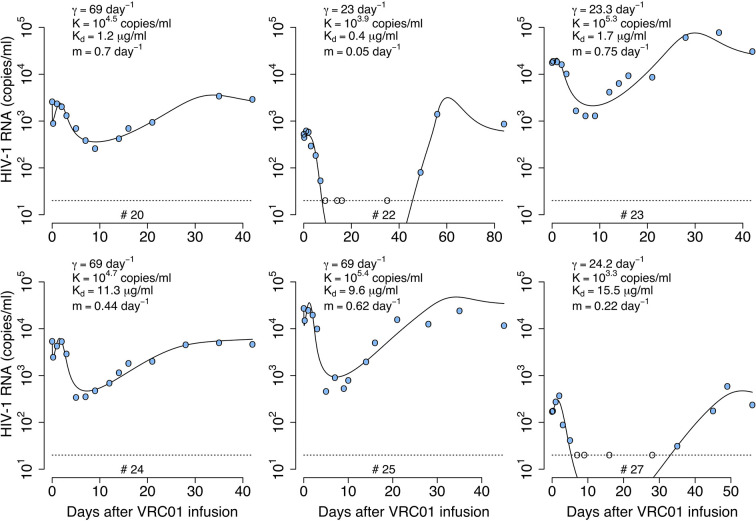
Best-fits of the phagocytosis-based logistic clearance model (PLCM), equation (19), to viral load data from participants receiving a single infusion of VRC01. Blue-filled and unfilled circles are the HIV-RNA over and below the limit of detection, respectively. Solid black lines are of best fit of the model, (*V* + *C*) in equation (19), to the data. Relevant parameter estimates for each participant are shown in each plot. Fixed parameter values are described in **Table 1**. See all parameter estimates in **Table S6**. Other parameters and initial values were derived by assuming the system was in steady state before VRC01 infusion.

We fit the model in equation (19) to the viral load data, estimating the parameters *γ*, *K*, *m, pT*(0)*, k_on_* and *ω*. In general, this model can capture the early fast viral decline and rebound in participant #20 and #24, the early viral rebound or plateau phase in participants #22 and #27, and the long-term viral decline and rebound in all four participants (see **Figure 9**). However, it misses several features in participants #23 and #25 (See individual parameter estimates in **Table S6**). This is demonstrated by the statistical improvement of the fits to the viral load only from participants #20, #22, #24 and #27 (ΔBIC>2.5 only by comparing the fits of this model to data from participants #20, #22, #24 and #27 with respect to the fits with all the previous models in **Table 2**).

The model predicts that the VRC01 dissociation constant for the sensitive virus $\left(K_{d_{s}}=\frac{k_{off}}{k_{on}}\right)$ is ~11.4 μg ml^-1^. This dissociation coefficient is higher than that estimated by the delayed neutralization model with one viral population and is also higher than the value estimated *in vitro*. Finally, the model predicts that the initial decay is due to the maximum clearance of the immune complexes *γ*, being between 1 and 4 times greater than the clearance rate of the free virus (*c* = 23 day^-1^), suggesting that VRC01 enhances the virus clearance.

#### Phagocytosis-Based Logistic Clearance Model With Two Viral Populations

Generalizing to two viral populations, the model becomes

(20) $$\begin{array}{c} \frac{dT}{dt}=\lambda -dT-\beta_{s}V_{s}T-\beta_{r}V_{r}T\\ \frac{dI_{s}}{dt}=f\beta_{s}V_{s}T-\hat{\delta }I_{s}^{\omega }\\ \frac{dI_{r}}{dt}=f\beta_{r}V_{r}T-\hat{\delta }I_{r}^{\omega }\\ \frac{dV_{s}}{dt}=pI_{s}-cV_{s}-k_{on_{s}}V_{s}A(t)+k_{off}C_{s}\\ \frac{dV_{r}}{dt}=pI_{r}-cV_{r}-k_{on_{r}}V_{r}A(t)+k_{off}C_{r}\\ \frac{dC_{s}}{dt}=k_{on_{s}}V_{s}A(t)-k_{off}C_{s}-\gamma \left(1-\frac{C_{p}}{K}\right)C_{s}\\ \frac{dC_{r}}{dt}=k_{on_{r}}V_{r}A(t)-k_{off}C_{r}-\gamma \left(1-\frac{C_{p}}{K}\right)C_{r}\\ \frac{dC_{p}}{dt}=\gamma \left(1-\frac{C_{p}}{K}\right)(C_{s}+C_{r})-mC_{p} \end{array}.$$

**Figure 10:**
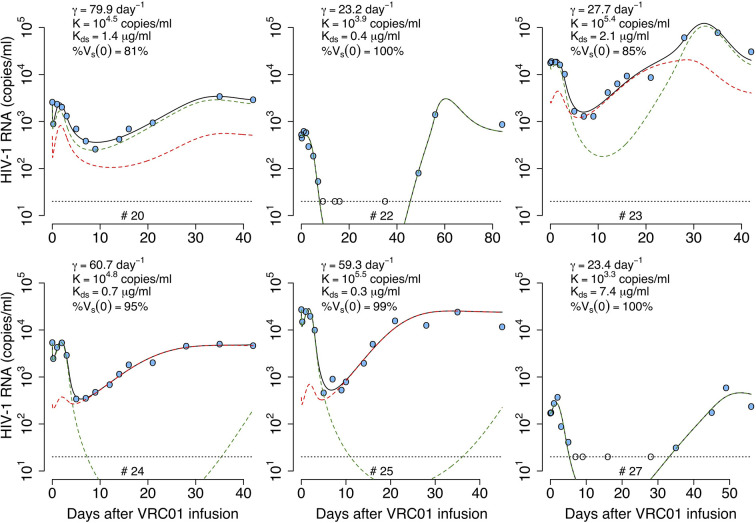
Best-fits of the two viral population phagocytosis-based logistic clearance model, equation (20), to viral load data from participants receiving a single infusion of VRC01. Blue-filled and unfilled circles are the HIV-RNA over and below the limit of detection, respectively. Solid black lines are the best-fit of the model, (*V_s_* + *V_r_* + *C_s_* + *C_r_*) in equation (20) to the data. Green and red dashed lines show the sensitive (*V_s_* +*C_s_*) and less sensitive (*V_r_ + C_r_*) virus concentration prediction of the model, respectively. Relevant parameter estimates for each participant are shown in each plot and all parameter estimates are given in **Table S7**. Fixed parameter values are described in **Table 1**. Other parameters and initial values were derived by assuming the system was in steady state before VRC01 infusion.

We fit this model to the data, estimating the parameters %*V_s_*(0), *γ*, *K*, *m*, *pT*(0), $k_{on_{s}},$ $k_{on_{r}}$ and *ω*. In general, this model is able to reproduce all the features of the viral load data from all participants well (**Figure 10**). However, the model only has better statistical support for the fits to participants #23, #24 and #25 compared to the best previous models (see **Table 2**). The model did not improve the fitting with respect to the one viral population version for participants #20, #22 and #27, but the estimated parameters reflect the same results for participants #20 and #22 (See **Table S7** for all best-fit estimates). For the best fits of participant #22 the model predicts that the sensitive virus population corresponds to almost 100% of the viral population, which is the same as having just a model with one viral population. Also, the model predicts that for participant #20, the dissociation constants for the sensitive and less-sensitive virus populations ( $K_{d_{s}}=\frac{k_{off}}{k_{on_{s}}}$ and $K_{d_{r}}=\frac{k_{off}}{k_{on_{r}}}$, respectively) are very similar, which also is equivalent to have a model with one viral population.

This model predicts a dissociation constant for the sensitive virus around 0.8 μg ml^-1^, also similar to the estimates from the delayed-neutralization model. The model also predicts that the sensitive virus corresponds to the majority (~90%) of the initial viral population. This value is relevant as it is similar to the 90% breadth of VRC01 estimated *in vitro* (32). As for the one viral population model, this model predicts VRC01 enhances the clearance of the virus by increasing the clearance rate of immune complexes up to ~4-fold, i.e., from 23 day^-1^ to 80 day^-1^. Finally, the model reproduces the rapid initial viral load decline and rebound, and estimates that captured immune complexes have a half-life of ~72 hrs.

To understand how the parameters drive the virus dynamics, we used the estimated parameter values for participant #24 to simulate the model and then did a sensitivity analysis by varying one parameter at a time. As presented in **Figure S2**, once the value of *γ* increases over the value of *c* = 23 day^-1^ a rapid, early virus decline is predicted by the model (**Figure S2A**). Since the following rebound depends on how quickly the captured immune complexes *C_p_* grow, the rebound is modulated by the parameters *K* and *m*. Thus, a higher and longer early viral rebound is predicted when *K* and *m* decrease (**Figures S2B, C**). Specifically, *K* must be smaller than the early viral load concentration, for an early viral rebound to appear. Since HIV complexed with VRC01 is assumed be neutralized and thus does not infect cells, if $K_{d_{s}}=\frac{k_{off}}{k_{on_{s}}}$ is sufficiently small compared to the VRC01 concentration the model predicts a long-term viral load decline (**Figure S2D**). However, if $K_{d_{s}}$ is close to or greater than the VRC01 concentration (i.e. $K_{d_{s}}>50$ μg ml^-1^) the viral decline slows down or is not seen because less virus is neutralized allowing viral replication and there is no early viral rebound because immune complexes are rarely formed (**Figure S2D**). A similar, non-responding behavior is seen if the fraction of the viral population sensitive to VRC01, %*V_s_*(0) is smaller than 50% (**Figure S2E**).

**Figure 11:**
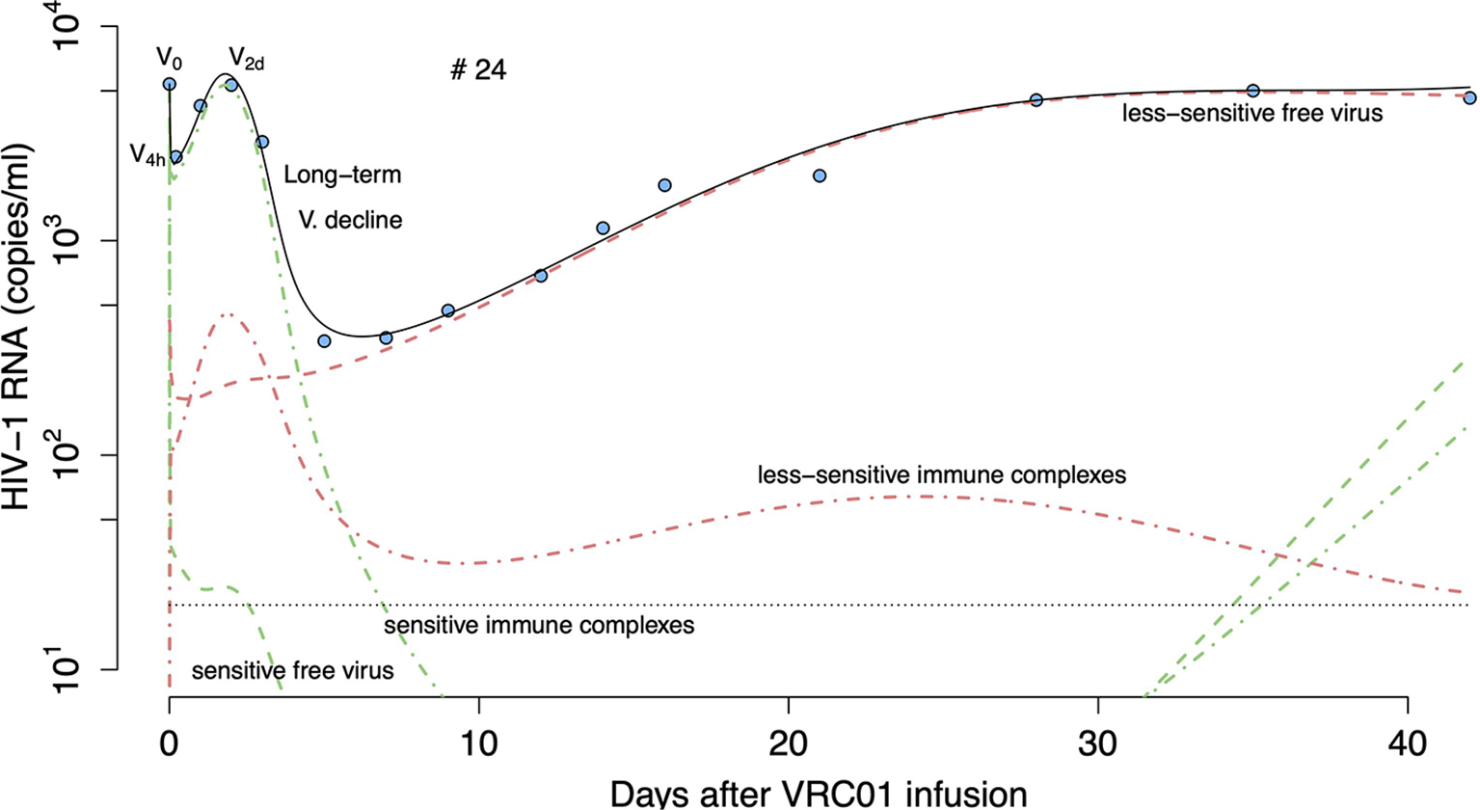
Prediction of the two viral population phagocytosis-based logistic clearance model, equation (20), for free virus and immune complexes in participant #24. Blue-filled circles are the HIV-RNA and the solid black line is the model predictions (*V_s_* + *V_r_* + *C_s_* + *C_r_*). Green and red dashed lines show the sensitive (*V_s_*) and less sensitive (*V_r_*) free virus concentration prediction. Red and green dotted-dashed lines present the complexes formed by VRC01 and the sensitive (*C_s_*) or less sensitive virus (*C_r_*).

As an illustration, **Figure 11** presents the predicted fate of the virus in volunteer #24 according to this model. At baseline, sensitive virus comprises 95% of the viral population (green dashed lines). During the first hours after VRC01 infusion, immune complexes are formed and are cleared faster than free virus, explaining the initial fast decay. As the phagocytic carrying capacity is reached, fewer immune complexes (green dot lines) are cleared producing a fast rebound over the next hours. Neutralization of virus by VRC01 leads to reduced *de novo* infection, and coupled with the death of already infected cells leads to the viral decay observed over the next couple of days. While the concentration of VRC01 is still high enough to affect the sensitive virus, a less VRC01-sensitive virus population can have selective advantage (red dashed lines) producing the final rebound in viral load.

### Comparison of All Models

We compared the ability of each model to explain the data by using model selection theory (39). In this approach, we computed the Bayesian information criterion (BIC) for the fit of each model to each participant and also by comparing a global Bayesian information criterion for all participants (BIC*_all_*), as described in the *Materials and Methods* section.

When comparing the BIC of the model for each participant (see **Table 2**) we found that the data from participants #20, #22 and #27 are better explained by the phagocytosis-based logistic clearance model with one viral population, while the data for participants #23, #24 and #25 are better explained by the same model but with two viral populations. From the estimates of the best model for each individual (**Table 3**) we found a dissociation constant for the sensitive virus around 0.9 μg ml^-1^, and that the sensitive virus corresponds to the ~95% of the initial viral population.

**Table 3:** Parameter estimate of the models with the lowest BIC values in **Table 2** for each individual.

	%*V_s_*(0)	log_10_(*pT*_0_)	$K_{d_{s}}$	$K_{d_{r}}$	*γ*	log_10_(*K*)	*m*	*ω*
	(-)	log_10_(1/ml/day)	(μg/ml)	(μg/ml)	(1/day)	log_10_(1/ml)	(1/day)	(-)
**#20**	–	8.3	1.1	–	75.9	4.5	0.8	2.5
**#22**	–	7.6	0.4	–	23.0	3.9	0.1	1.2
**#23**	0.85	8.3	2.1	28.9	27.7	5.4	0.3	1.3
**#24**	0.95	7.8	0.7	217.3	60.7	4.8	0.2	1.2
**#25**	0.99	8.3	0.3	91.5	59.3	5.5	0.3	1.2
**#27**	–	7.2	15.9	–	24.3	3.3	0.2	1.1
**Median**	0.95	8.1	0.9	91.5	43.5	4.7	0.2	1.2
**min**	0.85	7.2	0.29	28.9	23.0	3.3	0.1	1.1
**max**	0.99	8.3	15.9	217.3	75.9	5.5	0.8	2.5

To evaluate the robustness of the model selection process we fit each model again to ten viral load profiles constructed artificially by adding noise to participants’ viral load observations over the limit of detection. We assumed the noise was lognormally distributed with zero mean and a standard deviation of 0.2 log_10_ (**Figure S3**). Then, for each participant we computed the BIC of the models using the median of the sum of squares errors from the fits of each model to the ten profiles. As before, we found that the phagocytosis-based logistic clearance model better explains the data, with the model with one viral population better for #20, #22 and #27, and with two viral populations better for participants #23, #24 and #25 (**Tables S12, S13**).

When comparing the models using a global approach, we found that the model that best fit the data was the phagocytosis-based logistic clearance model with two viral populations (ΔBIC*_all_*>2.9, **Table 4**). As described in the previous section, although this model globally fits the data better, the estimated parameters for participants #20, #22 and #27 suggested that the sensitive viral population correspond to almost 100% of the final population or with very similar dissociation constant with respect to the less sensitive viral population, equivalent to have a model with a single viral population.

**Table 4:** Sum of squared error (SSE) and global Bayesian information criterion (BIC*_all_*) values.

ID	SSE					
	DNM		PSCM		PLCM	
	1 Viral pop.	2 Viral pops.	1 Viral pop.	2 Viral pops.	1 Viral pop.	2 Viral pops.
**#20**	0.24	0.21	0.53	0.13	0.06	0.06
**#22**	4.09	4.03	2.11	0.21	0.12	0.12
**#23**	0.60	0.43	0.80	0.51	0.50	0.21
**#24**	0.32	0.20	0.95	0.56	0.14	0.04
**#25**	0.71	0.51	1.75	1.45	0.89	0.35
**#27**	0.36	0.38	1.79	0.49	0.25	0.24
**Σ(SSE)**	6.32	5.77	7.93	3.36	1.97	1.02
***M_all_***	30	42	30	48	36	48
**BIC*_all_***	-87.6	-42.0	-68.3	-61.5	-160.0	**-162.9**
**ΔBIC*_all_***	75.3	120.9	94.6	101.4	2.9	0.0

DNM, Delayed-neutralization model; PSCM, Phagocytosis-based saturated clearance model; PLCM, Phagocytosis-based logistic clearance model. The lowest BIC*_all_*is given in bold type.

We finally compared the best model obtained by the global selection, with the equivalent model but assuming that the death rate of infected cells was constant (i.e. *ω* = 1). However, we found that the model with *ω* = 1 resulted in worse fits to the data (see **Table S11**).

## Discussion

A single infusion of 40mg/kg of VRC01 was able to reduce the viral load in chronically infected individuals more than 1-log (1). Since VRC01 is a bnAb with breadth of 90% *in-vitro*, one would expect that *in-vivo* it neutralizes the majority of virus strains preventing *de-novo* infection events. Thus, one would expect the virus dynamics during treatment with VRC01 would reflect the death rate of infected cells similarly to what is observed during treatment with reverse transcriptase inhibitors (RTIs). However, the observed kinetics after VRC01 passive administration are quite distinct from those observed after initiation of treatment with RTIs (1, 18, 21). The data shows that the major reduction in viral load occurs after a delay of about 2-3 days, which is longer than the one observed after initiation of antiretroviral therapy (9, 10, 18, 21, 40). If one assumes the data is accurate, in three of the individuals the virus declined rapidly during the initial 4 hours and then rebounded to baseline by day 1, and in two other treated individuals, the early decline was not captured but an initial viral increase to above baseline was observed.

The measured serum concentration of VRC01 decayed in a biphasic manner, similar to the dynamics observed with other monoclonal antibodies (16). Therefore, we fit the antibody concentration data to a two-compartment pharmacokinetics model (representing the VRC01 concentration in plasma and tissues) where the first phase decay occurs as the antibody is distributed from blood to tissue, and the second phase represents antibody elimination from the body (17). We developed closed form solutions for the serum VRC01 concentration in both compartments and showed that the volume of the compartments do not affect the VRC01 dynamics in the first compartment (plasma), but it affect its concentration in second compartment (tissues). Pharmacokinetic analysis showed that infusion of VRC01 in viremic and aviremic individuals did not have significant differences. This result suggests that the concentration of VRC01 was sufficiently high that the binding of VRC01 to the virus in infected individuals did not noticeable affect the serum VRC01 concentration during the first 6 weeks following infusion. Our pharmacokinetic model predicts that VRC01 in infected individuals is eliminated with a half-life of 7.1 days. This value is smaller than the 12 days estimated previously in infected individuals using a non-compartmental PK analysis (1, 8). However, our PK model includes transport of the Ab between blood and tissue, codifying explicitly the mechanisms for both the first (distribution) and second (elimination) decay phases observed in the VRC01 concentration time course data (17).

To explain the viral dynamics in chronically infected individuals after a single infusion of VRC01 we developed models by modifying the standard model of virus dynamics in (19, 20). Since Lynch et al. (1) detected virus resistant VRC01 in 2 individuals at baseline, who were not modeled here as their viral load did not decline, and in the six individuals studied here a virus population less sensitive to VRC01 a month after infusion, we generalized each model to include two viral populations with one more sensitive to VRC01 than the other, assuming that the observed viral load reflects the sum of the two viral populations. Here we presented three different mathematical models, with one or two viral populations, that could explain the observed viral load data. The first model assumed that VRC01 neutralizes the virus after a delay. However, this model did not explain the mechanism behind the initial delay. The other two models assumed that the mechanism behind the “delay” has to do with the capacity of VRC01 to opsonize HIV-1 and increase the rate of phagocytosis to clear the virus, reflected in the initial rapid decline in the viral load after VRC01 infusion. To capture that mechanism these models included an explicit term for the formation of immune complexes and explored different approaches regarding their clearance. The best-fit model assumed that clearance of immune complexes comprises a two-step process that includes capture of immune complexes on the surface of phagocytes that have a maximum carrying (binding) capacity followed by the internalization/degradation of the complexes, which then allows the phagocytes to bind additional immune complexes. Based on BIC, we found that this model, with capture and internalization/degradation of immune complexes, consistently explained the data better for each participant than the other models we examined; with one viral population for participants #20, #22, and #27, and two viral populations for participants #23, #24, and #25.

One of the main implications of this result is that it suggests that VRC01, through the formation of HIV-VRC01 immune complexes, has the capacity to enhance the clearance of the virus up to 3–fold compared to estimates of free virus clearance. This process is necessary to explain the rapid viral decline during the initial hours after VRC01 infusion. This result concurs with early studies that demonstrated rhesus macaques receiving a continuous infusion of HIV and high titers of virus-targeted antibodies experienced an enhancement in virus clearance of up to 4-fold in the presence of the antibodies (41). It also agrees with a recent study in humanized mice showing the ability of 3BNC117, a bnAb also targeting the CD4 binding site, to enhance the clearance of mAb-opsonized virus from the circulation (42). The second main implication is that the best model suggests that the clearance of VRC01-HIV immune complexes behaves as a phagocytosis-like process, but predicts that this process is constrained during immune complex capture and possibly internalization/degradation of HIV-VRC01 immune complexes. This limitation is necessary to explain the viral rebound before the major viral load reduction.

It has been shown that besides neutralization antibodies can activate phagocytic cells *in-vivo* following the opsonization of antigens by antibodies, particularly IgG_1_, the isotype of VRC01, and the binding of their Fc region to Fcγ receptors on the surface of phagocytes (43). Previous *in-vitro* studies have suggested that monocyte-derived macrophages use Fcγ receptors (FcγR) to phagocytose and clear HIV-IgG complexes (44). Additionally, *in-vivo* studies in humanized mice have revealed that viral opsonization by mAbs targeting the CD4bs enhanced their clearance by circulating or tissue-resident cells expressing FcγR (42). More recently, a study has shown that the majority of effectors cells expressing FcγR in human mucosal tissues are phagocytes, with significant phagocytic activity (45). Thus, our results, along with the findings mentioned above, suggest that effector mechanisms like phagocytosis of VRC01-HIV immune complexes are likely to clear virus-mAb immune complexes, and if it enhances the clearance of the virus it can explain the early virus dynamics observed after VRC01 infusion.

Our results also suggest that in order to have the initial viral load rebound (or plateau phase) at day 1, phagocytic capacity has to be constrained. A similar rebound over baseline was present in 10 of 18 infected participants one day after receiving a single infusion of 3BNC117 (3), and in 7 of 13 participants receiving 30 mg/kg of the bnAb 10-1074 (46). Interestingly, our model can recapitulate the viral loads of individuals receiving 3BNC117, suggesting the same mechanism may drive those early viral rebounds (manuscript in preparation). The nature behind this phagocytic impairment is unclear, but it might be due either to the loss of Fc receptors by internalization, high amounts of circulating immune complexes prior to VRC01 infusion that may block access to FcγRs (37, 38) or due to a finite capacity to internalize and degrade immune complexes, among other reasons. In any event, our model predicts that phagocytic capacity returns with a t_1/2_ of ~3 days. In lymphoid tissues, where the majority of HIV-1 infection events occur, FcγR expressing macrophages and neutrophils are present (45). However, in HIV-1 infected individuals the capacity of FcγR-expressing phagocytes can be impaired (47–50) as the expression of FcγRs is significantly reduced in chronic infection (36). This reduction in FcγR expression may make it easier to saturate the cellular phagocytic capacity. Furthermore, phagocytosis can be impaired in the presence of HIV proteins, as they might prevent the recruitment of key proteins into the phagocytic cup (51), or perturb phagosome formation (52) and fusion to lysosomes (53). Thus, although in the absence of HIV-1 phagocytosis of viruses or bacteria by neutrophils or macrophages may take from several minutes (54) to a couple of hours (55), our results along with the studies referenced above may indicate that the capacity of this process might be impaired and the degradation activity delayed in chronic HIV-1 infection.

Our model predicts that VRC01 has a maximum *in-vivo* dissociation constant for the sensitive virus $\left(\frac{k_{off_{s}}}{k_{on_{s}}}\right)$ of 0.9 μg/ml. Since VRC01 has *in-vitro* neutralization potency with IC_50_ estimates similar to the dissociation constant estimated here (32), our findings indicate that *in-vitro* estimates of neutralization potency might be surrogates for *in-vivo* virologic effects (1). Furthermore, in agreement with neutralization and sequence analyses (1), the model predicts that in several participants a second less VRC01-sensitive viral population might be selected, and that the majority of virus in the observed viral rebound might come from this second population. In those cases, the sensitive virus was around 95% of the initial virus population. This fraction is relevant as it might be related to the previous estimate of the 90% breadth of VRC01 (32).

The phagocytosis-based logistic clearance model included the reversible formation and dissociation of sensitive-virus/VRC01 neutralized complexes. As in the model of Lu et al. (15), this is the simplest model of viral neutralization in which only a single antibody binds to each virion, leading to its neutralization. In reality, a number of antibodies can bind to a virion, but as VRC01 is in great excess this extra binding does not appear to affect the free antibody concentration. However, these extra antibodies could bind free Fc receptors and more detailed models of phagocytosis and viral neutralization would be needed to take this into account.

Finally, our model predicts that the death rate of infected cells is not constant, as traditionally modeled to fit virus dynamics (10, 19–21), but it depends on the density of infected cells, codified by the parameter *ω*, as first introduced by Holte et al. (28). We found that assuming a constant rate of infected cell death (*ω* = 1) the fits of the model to the data are significantly degraded, and that viral rebound cannot be reproduced accurately. Interpretations of this non-linear, infected cell-density dependent rate may suggest that cytolytic processes that are activated by an increased number of infected cells may be present, such as CD8+ T cell mediated cell killing (28, 56).

In conclusion, we have compared several models to understand the mechanism behind the virus dynamics observed in chronically infected participants treated with a single infusion of VRC01. From our comparison, the best model suggests that a single infusion of VRC01 induces an enhancement of virus clearance by a phagocytic mechanism that rapidly clears VRC01-HIV complexes. Our analysis also suggests that this phagocytic mechanism is limited, and that VRC01-HIV complex clearance might slow as the process becomes saturated, possibly due to internalization or blocking of Fc receptors. This explains the initial fast decay and rebound in the plasma viral load observed after VRC01 infusion. The long-term viral decline is due to neutralization of the virus by VRC01 with similar efficacy estimated by *in-vitro* methods. However, selection pressure may lead to the outgrowth of a less-susceptible virus population to VRC01 with lowered neutralization potency reflected in the viral load final rebound.

## Materials and Methods

### Clinical Data

We fit our proposed models to data from the VRC 601 single-site, phase 1, open-label, dose escalation study conducted at the NIH Clinical Center by the VRC Clinical Trials Program, NIAID, NIH (ClinicalTrials.gov NCT 01950325) (1, 8).

For the VRC01 pharmacokinetic analysis we used two sets of data. The first came from the six HIV-1 infected individuals who had a significant decrease in their viral load after a single VRC01 infusion of 40 mg/kg. We did not use the VRC01 concentration data from the two infected individuals who did not respond to the mAb as we wanted to know if the binding of VRC01 to HIV-1 significantly changed the VRC01 serum concentration. The data comprises serum VRC01 concentration measurements collected before infusion and at 0, 1, 2, 4, and 24 hours, and days 2, 7, 14, 21, 28, 35, 42, 49, 56, and 84 after infusion (1). The second set of data comes from the three-aviremic participants who received one or two VRC01 infusions of 40 mg/kg. Two of them were infused at days 0 and 28, and samples were collected at 0, 1, 2, 4, 8, 12, and 24 hours, as well as 2, 7, 14, 21 and 28 days after each infusion (8). One of the three-aviremic individuals completed only one infusion following the same collection of data until day 28 after VRC01 administration. VRC01 serum concentration quantification was performed in 96-well plates on a Beckman Biomek–based automation platform using the anti-idiotype mAb 5C9. VRC01 concentration was undetectable at levels smaller than 0.098 μg/ml.

For the viral kinetic model analysis, we used HIV RNA plasma samples from six HIV-1 infected participants after VRC01 infusion. Measurements were performed by the NIH Clinical Center using the standard diagnostic assay COBAS AmpliPrep/COBAS TaqManHIV-1 Test, version 2.0. We used samples collected at baseline, 4 hours, as well as days 1, 2, 3, 5, 7, 14, 16, 21, 28, 35 and 42 after VRC01 administration for all participants, but also days 49, 56, and 84 for participants #22 and #27 (1), for a total of 85 data points from all infected participants who responded to VRC01 combined (participants #20, #22, #23, #24, #25 and #27). Virus load was undetectable at concentrations below 20 copies/ml.

### Pharmacokinetic Model Fitting

We separately fit the two-compartment PK model solution in Eqs. (2) and (4) to the VRC01 serum concentration measurements from the six HIV-1 infected participants and from three aviremic, presumably non-infected, volunteers infused with 40 mg/kg of VRC01 using a non-linear least-squares approach. We estimated the parameters *k*_0_, *k*_12_ and *k*_21_.

To convert from units of [μg/ml], we assumed that VRC01 has the IgG molecular weight of 160 kDa. Thus, we computed a conversion factor as follows,

$$160\;\text{kDa}=1.6\times 10^{5}\text{g}/\text{mol}\times 6.022\times 10^{-23}\text{mol}/\text{molecule}\times 10^{6}\mu \text{g}/\text{g}=2.7\times 10^{-13}\mu \text{g}/\text{molecule}.$$

### Viral Kinetic Model Fitting and Selection

Using the best-fit of the VRC01 concentration in blood for each infected individual as *A*(*t*), we then fit the viral kinetic models with one or two viral populations to the plasma HIV RNA data of the six participant that responded to VRC01.

Because the viral production rate *p* cannot be estimated from the standard viral dynamics model using only viral load data (57, 58) we redefined the variables in all the virus dynamics models so that $\hat{I}=pI,$ and $\hat{T}=pT,$ so we can re-write, without loss of generality, the equations for *T*, *I* and *V* as,

(21) $$\begin{array}{l} \frac{d\hat{T}}{dt}=\hat{\lambda }-d\hat{T}-\beta V\hat{T}\\ \frac{d\hat{I}}{dt}=\beta V\hat{T}-\tilde{\delta }\hat{I}\omega^{\;}\\ \frac{dV}{dt}=\hat{I}-cV \end{array}$$

Where $\tilde{\delta }=p^{1-\omega }\hat{\delta }$ and $\hat{\lambda }=p\lambda$. We did the same for the models with two viral populations.

For each participant, we determined a parameter set minimizing the sum of squared error function $\sum_{i}\left[log(y_{i})-\log\;f\;(t_{i}\right){]}^{2},$ where *f*(*t_i_*) represents the numerical solution for the viral load at time *t_i_* derived from the model, and *y_i_* represents the measured HIV RNA value at time *t_i_*. For the models with an explicit term for immune complexes, the model viral loads were calculated as *V_s_* + *C_s_*, and *V_s_* + *C_s_* + *V_r_* + *C_r_*, for the case of one and two viral populations, respectively. Otherwise, the model viral loads were calculated as *V_s_*, and *V_s_* + *V_r_*. For participants #22 and #27, in which the viral loads fell below the limit of detection, we fit each model to the data by minimizing the following adjusted sum of squared error, that takes into consideration censored data:

(22) $$SSE=\sum_{i\in I_{v>LD}}{\left[\text{log}(\gamma_{i})-\text{log}\;f(t_{i})\right]}^{2}+\sum_{i\in I_{v<LD}}-\text{log}[F_{LN}\{LD,\text{log}\;f(t_{i}),\sigma_{i}\}]$$

where *F_LN_* represents the lognormal cumulative distribution at the limit of detection level with mean *f*(*t_i_*) and variance $\sigma_{i}^{2}$ (where the negative symbol in the censored-data-term in equation (22) is used to minimize the function, and $F_{LN}\{LD,\log\;f(t_{i}),\sigma_{i}\}=\frac{1}{\sqrt{2\pi \sigma_{i}^{2}}}\int_{-\infty }^{LD}e^{-(u-\log\;f(t_{i}){)}^{2}}du)$ (59). The sets *I_v_*_>_ *_LD_* and *I_v_*_<_ *_LD_* represent the sets of HIV RNA measurements above and below the limit of detection, respectively. Since there was no additional single copy assay data to obtain information about the values below the limit of detection, we used an approach for censored data with a model for $\sigma_{\text{i}}^{2}$ fit to single-copy assay (SCA) data proposed elsewhere (60, 61).

Fixed parameter values used are the target cell death rate *d* = 0.01 day^-1^ (31) and the virus clearance rate c = 23 day^-1^ (30). Since the death rate of infected cells is density-dependent, we computed the value of $\tilde{\delta }$ by assuming that the initial infected cell death rate $\tilde{\delta }\hat{I}{\left(0\right)}^{\omega -1}=1.5\text{da}{\text{y}}^{-1}$ [close to the maximum constant death rate estimates during ART (18)], so that $\tilde{\delta }=\frac{1.5}{\hat{I}{\left(0\right)}^{\omega -1}}$, where the value of *ω* is fitted, and $\hat{I}(0)$ is obtained using the steady-state assumption at the beginning of VRC01 infusion as described below. We used the maximum estimate of *δ* from (18) as we expect the density of infected cells and hence $\delta ={\tilde{\delta }\hat{I}}^{\omega -1}$ to decrease with time. In the models that have binding and dissociation of VRC01 to the virus, we used a dissociation rate of 2.75 day^-1^ (32, 33). For all models, we assumed that the initial values of the variables in the model correspond to the participant being at steady state (set-point of chronic infection) before the VRC01 infusion. In the case of models with two viral populations, we assumed that the initial value of the sensitive viral population to VRC01, *V_s_*(0), was equal to the measured baseline viral load data multiplied by the estimated fraction $\frac{V_{S}(0)}{V(0)}$, from the fitting procedure. During the fitting we estimated the fraction $\frac{V_{S}(0)}{V(0)}$. Similarly, $V(0)\left[1-\frac{V_{S}(0)}{V(0)}\right].$ We assumed *C_s_*(0) = *C_r_*(0) = 0. We calculated the initial values of infected cells from the steady-state equations before the infusion of VRC01 with forms ${\hat{I}}_{s}(0)=cV_{s}(0)$ and ${\hat{I}}_{r}(0)=cV_{r}(0)$. Also using the initial values presented above we estimated the production rate of target cells as $\hat{\lambda }=d\hat{T}(0)+\beta_{s}V_{s}(0)\hat{T}(0)+\beta_{r}V_{r}(0)\hat{T}(0)$ (we also performed fits assuming $\hat{\lambda }=d\hat{T}(0)$ but obtained equivalent results), with infectivity rates for the sensitive and less-sensitive virus $\beta_{S}=\frac{{\tilde{\delta }\hat{I}}_{s}{\left(0\right)}^{\omega }}{fV_{s}(0)\hat{T}(0)}$ and $\beta_{r}=\frac{\tilde{\delta }{\hat{I}}_{r}{\left(0\right)}^{\omega }}{fV_{r}(0)\hat{T}(0)}.$

We used the Differential Evolution package in R to find initial parameter estimates and improved them using the L-BFGS-B algorithm in the R-optim package. We performed model selection for each participant using the Bayesian information criterion $\left(BIC=n\;\log\left(\frac{SSE}{n}\right)+M\;\log(n)\right)$, where *M* is the number of estimated parameters for the model and *n* is the number of data points. Then, we compared the *BIC* values from different models and computed the values of Δ*BIC* by subtracting the minimum *BIC* from each model’s *BIC*. We assumed there is substantial evidence against models with higher *BIC* if their corresponding Δ*BIC* > 2 (39). We also performed model selection globally for all participants by computing $BIC_{all}=n_{all}\;\log\left(\frac{SSE_{all}}{n_{all}}\right)+M_{all}\;\log(n_{all})$, where *SSE_all_* is the sum of squared error of the fits from all participants viral load data using a specific model, *M_all_* is the total number of parameter estimated for all participants, i.e. *M_all_* = *M* × 6, and *n_all_* the total number of data points (in this case, *n_all_* = 85). We computed the values of Δ*BIC_all_* by comparing the *BIC_all_* of each model with the minimum *BIC_all_* from all models. We also assumed a substantial evidence against the models with higher *BIC_all_* if their corresponding Δ*BIC_all_* > 2.

## Data Availability Statement

Publicly available datasets were analyzed in this study. This data can be found here: Lynch, R. M. et al. Sci Trans Med 7, 319ra206 (2015).

## Ethics Statement

The studies involving human participants were reviewed and approved by Los Alamos National Laboratory Institutional Review Board. The patients/participants provided their written informed consent to participate in this study.

## Author Contributions

EC and AP conceived the study and developed the models. EC assembled data, wrote all code, performed all calculations and derivations, ran the models, and analyzed output data. AP and EC wrote the manuscript. All authors contributed to the article and approved the submitted version.

## Funding

Portions of this work were one under the auspices of the U.S. Department of Energy under contract 89233218CNA000001. This study was supported by grants from the National Institutes of Health, R01 AI150500 (EC) and R01 AI028433, R01 OD011095 and P01 AI131365 (AP). The funders had no role in study design, data collection and analysis, decision to publish, or preparation of the manuscript.

## Conflict of Interest

The authors declare that the research was conducted in the absence of any commercial or financial relationships that could be construed as a potential conflict of interest.

## Publisher’s Note

All claims expressed in this article are solely those of the authors and do not necessarily represent those of their affiliated organizations, or those of the publisher, the editors and the reviewers. Any product that may be evaluated in this article, or claim that may be made by its manufacturer, is not guaranteed or endorsed by the publisher.
