# Supplementary material for: Modeling HIV-1 Within-Host Dynamics After Passive Infusion of the Broadly Neutralizing Antibody VRC01
Source: Front Immunol. 2021 Aug 31;12:710012. doi: 10.3389/fimmu.2021.710012 (PMC8438300; doi:10.3389/fimmu.2021.710012)
Supplement: Supplementary file 1 [file DataSheet_1.docx]

Supplementary Material

Modeling HIV-1 within-host dynamics after passive infusion of the broadly neutralizing antibody VRC01

E. Fabian Cardozo^1^ and Alan S. Perelson^2*^

^1^Vaccine and Infectious Disease Division, Fred Hutchinson Cancer Research Center, Seattle, WA, USA.

^2^Theoretical Biology and Biophysics, Los Alamos National Laboratory, Los Alamos, NM, USA.

# * Correspondence: Corresponding Author Alan S. Perelson, MS K710, Los Alamos National Laboratory, Los Alamos, NM, USA. Email: [asp@lanl.gov](mailto:asp@lanl.gov).

# Supplementary figures

Figure S1. Fitting of the model in equation (17) to viral load data from subjects receiving a single infusion of VRC01. Blue and white circles are the HIV-RNA over and below the limit of detection, respectively. Solid black lines are the prediction of best fit of the model in equation (17) to the data (*V* + *C*). Fixed parameter values are described in Table 1. See all parameters estimates in Table S4. Other parameters and initial values were taken by assuming the system was in steady state before VRC01 infusion.

**Figure S2. Local sensitivity analysis of the phagocytosis-based logistic clearance model.** Sensitivity analysis was performed by simulating the model using the best-fit parameter estimates for participant #24 and varying the following parameters, only one at a time: **a.** maximum clearance of immune complexes $\gamma$, **b.** internalization and degradation rate $m$ of captured complexes, **c.** carrying capacity for immune complexes internalization, $K$, **d.** VRC01 dissociation constant for the sensitive virus $\left( K_{d}=\frac{k_{off}}{k_{on}} \right)$, **e.** Initial fraction of VRC01 sensitive virus %$V_{s}(0)$ and **f.** $\omega$, the parameter that quantifies the density dependent rate of infected cells.

**Figure S3. Artificial viral load data from each individual to evaluate the robustness of model selection.** Red lines represent the original data and gray lines are the data created by adding noise to all participants’ viral load observations over the limit of detection assuming the noise is lognormally distributed with a zero mean and a standard deviation of 0.2 log_10_.

# Supplementary tables

**Table S1. Parameters estimates from the best fits of the pharmacokinetics model to the serum VRC01 concentration. The value of *Vol*_2_ is computed assuming plasma volume, *Vol_1_*=3L, and *Vol*_2_=*k*_12_*Vol*_1_/*k*_21_.**

|  | ***k*_0_** | ***k*_12_** | ***k*_21_** | ***Vol*_2_** |
| --- | --- | --- | --- | --- |
|  | (1/day) | (1/day) | (1/day) | (ml) |
| **#20** | 0.10 | 0.80 | 0.14 | 17558 |
| **#22** | 0.15 | 0.71 | 0.20 | 10505 |
| **#23** | 0.12 | 0.61 | 0.11 | 16449 |
| **#24** | 0.10 | 0.64 | 0.13 | 14481 |
| **#25** | 0.08 | 0.56 | 0.10 | 16939 |
| **#27** | 0.08 | 1.05 | 0.21 | 14861 |
| **Median** | 0.09 | 0.68 | 0.13 | 15655 |
| **min** | 0.08 | 0.56 | 0.10 | 10505 |
| **max** | 0.15 | 1.05 | 0.21 | 17558 |

**Table S2. Parameters estimates from the best fits of the delayed-neutralization model with one viral population to the viral load data.**

|  | ***τ*** | **log_10_(*pT*_0_)** | ***K_d_*** | **log_10_(*V*_0_)** | ***ω*** | **BIC** | **SSE** |
| --- | --- | --- | --- | --- | --- | --- | --- |
|  | (days) | log_10_(1/ml/day) | (μg/ml) | log_10_(1/ml) | (-) |  |  |
| **#20** | 2.5 | 6.8 | 0.4 | 3.4 | 2.0 | -39.1 | 0.24 |
| **#22** | 3.0 | 7.0 | 0.1 | 2.8 | 1.3 | -4.0 | 4.09 |
| **#23** | 1.6 | 8.9 | 15.5 | 4.3 | 1.2 | -34.8 | 0.60 |
| **#24** | 2.2 | 7.7 | 6.1 | 3.6 | 1.4 | -44.0 | 0.32 |
| **#25** | 1.8 | 7.7 | 2.2 | 4.3 | 1.3 | -32.2 | 0.71 |
| **#27** | 2.4 | 6.4 | 1.3 | 2.3 | 1.3 | -37.9 | 0.36 |
| **Median** | 2.3 | 7.4 | 1.7 | 3.5 | 1.3 |  |  |
| **min** | 1.6 | 6.4 | 0.1 | 2.3 | 1.2 |  |  |
| **max** | 3.0 | 8.9 | 15.5 | 4.3 | 2.0 |  |  |

**Table S3. Parameters estimates from the best fits of the delayed-neutralization model with two viral populations to the viral load data.**

|  | ***τ*** | **%*V_s_*(0)** | **log_10_(*pT*_0_)** | ***K_ds_*** | ***K_dr_*** | **log_10_(*V*_0_)** | ***ω*** | **BIC** | **SSE** |
| --- | --- | --- | --- | --- | --- | --- | --- | --- | --- |
|  | (days) | (-) | log_10_(1/ml/day) | (μg/ml) | (μg/ml) | log_10_(1/ml) | (-) |  |  |
| **#20** | 2.5 | 0.99 | 5.3 | 0.02 | 2.2 | 3.3 | 2.2 | -35.7 | 0.21 |
| **#22** | 3.0 | 0.78 | 6.5 | 0.02 | 0.1 | 2.8 | 1.3 | 1.1 | 4.03 |
| **#23** | 2.2 | 0.98 | 7.8 | 0.7 | 237.1 | 4.3 | 1.3 | -34.2 | 0.43 |
| **#24** | 2.4 | 0.95 | 7.8 | 1.1 | 335.4 | 3.7 | 1.2 | -45.8 | 0.20 |
| **#25** | 1.8 | 0.99 | 8.3 | 0.4 | 131.3 | 4.4 | 1.2 | -31.7 | 0.51 |
| **#27** | 2.5 | 0.63 | 6.5 | 2.3 | 2.8 | 2.3 | 1.3 | -32.0 | 0.38 |
| **Median** | 2.4 | 0.96 | 7.2 | 0.6 | 67.0 | 3.5 | 1.3 |  |  |
| **min** | 1.8 | 0.63 | 5.3 | 0.02 | 0.1 | 2.3 | 1.2 |  |  |
| **max** | 3.0 | 0.99 | 8.3 | 2.3 | 335.4 | 4.4 | 2.2 |  |  |

**Table S4. Parameters estimates from the best fits of the phagocytosis-based saturated clearance model with one viral population to the viral load data.**

|  | **log_10_(*pT*_0_)** | ***K_d_*** | ***γ*** | **log10(*K*)** | ***ω*** | **BIC** | **SSE** |
| --- | --- | --- | --- | --- | --- | --- | --- |
|  | log_10_(1/ml/day) | (μg/ml) | (1/day) | log_10_(1/ml) | (-) |  |  |
| **#20** | 6.4 | 0.1 | 23.0 | 4.8 | 2.5 | -28.7 | 0.53 |
| **#22** | 7.2 | 0.1 | 23.4 | 2.4 | 1.6 | -13.3 | 2.11 |
| **#23** | 7.9 | 0.5 | 23.0 | 4.3 | 2.1 | -30.4 | 0.80 |
| **#24** | 6.3 | 0.1 | 23.5 | 7.5 | 2.2 | -27.8 | 0.95 |
| **#25** | 6.7 | 0.1 | 25.4 | 7.6 | 1.5 | -18.7 | 1.75 |
| **#27** | 6.6 | 0.9 | 23.0 | 2.0 | 1.7 | -15.6 | 1.79 |
| **Median** | 6.6 | 0.1 | 23.2 | 4.5 | 1.9 |  |  |
| **min** | 6.3 | 0.1 | 23.0 | 2.0 | 1.5 |  |  |
| **max** | 7.9 | 0.9 | 25.4 | 7.6 | 2.5 |  |  |

**Table S5. Parameters estimates from the best fits of the phagocytosis-based saturated clearance model with two viral populations to the viral load data.**

|  | **%*V_s_*(0)** | **log_10_(*pT*_0_)** | ***K_ds_*** | ***K_dr_*** | ***γ_s_*** | ***γ_r_*** | **log_10_(*K*)** | ***ω*** | **BIC** | **SSE** |
| --- | --- | --- | --- | --- | --- | --- | --- | --- | --- | --- |
|  | (-) | log_10_(1/ml/day) | (μg/ml) | (μg/ml) | (1/day) | (1/day) | log_10_(1/ml) | (-) |  |  |
| **#20** | 0.93 | 7.7 | 0.2 | 3.0 | 101.0 | 1.0 | 6.0 | 1.3 | -39.4 | 0.13 |
| **#22** | 0.85 | 7.3 | 0.1 | 0.2 | 106.9 | 0.8 | 4.4 | 1.1 | -38.0 | 0.21 |
| **#23** | 0.82 | 8.5 | 1.7 | 6.1 | 110.9 | 1.6 | 6.5 | 1.2 | -29.0 | 0.51 |
| **#24** | 0.89 | 8.2 | 0.03 | 10.1 | 65.8 | 1.7 | 8.0 | 1.2 | -27.6 | 0.56 |
| **#25** | 0.66 | 6.9 | 0.01 | 0.2 | 23.8 | 23.8 | 8.9 | 1.5 | -13.3 | 1.45 |
| **#27** | 0.69 | 6.9 | 0.02 | 2.6 | 107.2 | 1.6 | 4.6 | 1.2 | -25.7 | 0.49 |
| **Median** | 0.84 | 8 | 0.04 | 2.8 | 103.9 | 1.6 | 6.2 | 1.2 |  |  |
| **min** | 0.66 | 7 | 0.01 | 0.2 | 23.8 | 0.8 | 4.4 | 1.1 |  |  |
| **max** | 0.93 | 9 | 1.70 | 10.1 | 110.9 | 23.8 | 8.9 | 1.5 |  |  |

**Table S6. Parameters estimates from the best fits of the phagocytosis-based logistic clearance model with one viral population to the viral load data.**

|  | **log10(*pT*_0_)** | ***K_d_*** | ***γ*** | **log_10_(*K*)** | ***m*** | ***ω*** | **BIC** | **SSE** |
| --- | --- | --- | --- | --- | --- | --- | --- | --- |
|  | log_10_(1/ml/day) | (μg/ml) | (1/day) | log_10_(1/ml) | (1/day) | (-) |  |  |
| **#20** | 8.3 | 1.1 | 75.9 | 4.5 | 0.8 | 2.5 | -54.0 | 0.06 |
| **#22** | 7.6 | 0.4 | 23.0 | 3.9 | 0.1 | 1.2 | -50.9 | 0.12 |
| **#23** | 8.5 | 1.7 | 23.2 | 5.3 | 0.8 | 1.8 | -34.8 | 0.50 |
| **#24** | 8.6 | 12.1 | 99.5 | 4.7 | 0.4 | 1.4 | -53.4 | 0.14 |
| **#25** | 9.2 | 10.6 | 104.6 | 5.4 | 0.5 | 1.3 | -26.0 | 0.89 |
| **#27** | 7.2 | 15.9 | 24.3 | 3.3 | 0.2 | 1.1 | -40.4 | 0.25 |
| **Median** | 8.5 | 11.4 | 61.9 | 5.0 | 0.4 | 1.3 |  |  |
| **min** | 7.2 | 1.7 | 23.2 | 3.3 | 0.2 | 1.1 |  |  |
| **max** | 9.2 | 15.9 | 104.6 | 5.4 | 0.8 | 2.5 |  |  |

**Table S7. Parameters estimates from the best fits of the phagocytosis-based logistic clearance model with two viral populations to the viral load data.**

|  | **%*V_s_*(0)** | **log_10_(*pT*_0_)** | ***K_ds_*** | ***K_dr_*** | ***γ*** | **log_10_(*K*)** | ***m*** | ***ω*** | **BIC** | **SSE** |
| --- | --- | --- | --- | --- | --- | --- | --- | --- | --- | --- |
|  | (-) | log_10_(1/ml/day) | (μg/ml) | (μg/ml) | (1/day) | log_10_(1/ml) | (1/day) | (-) |  |  |
| **#20** | 0.81 | 7.8 | 1.4 | 1.4 | 79.9 | 4.5 | 0.8 | 2.2 | -49.7 | 0.06 |
| **#22** | 1.00 | 7.6 | 0.4 | 0.8 | 23.2 | 3.9 | 0.1 | 1.2 | -45.3 | 0.12 |
| **#23** | 0.85 | 8.3 | 2.1 | 28.9 | 27.7 | 5.4 | 0.3 | 1.3 | -42.1 | 0.21 |
| **#24** | 0.95 | 7.8 | 0.7 | 217.3 | 60.7 | 4.8 | 0.2 | 1.2 | -68.7 | 0.04 |
| **#25** | 0.99 | 8.3 | 0.3 | 91.5 | 59.3 | 5.5 | 0.3 | 1.2 | -34.6 | 0.35 |
| **#27** | 0.62 | 7.0 | 0.9 | 14.6 | 23.6 | 3.3 | 0.1 | 1.1 | -36.1 | 0.24 |
| **Median** | 0.90 | 7.8 | 0.8 | 21.8 | 43.5 | 4.7 | 0.2 | 1.2 |  |  |
| **min** | 0.62 | 7.0 | 0.29 | 0.8 | 23.2 | 3.3 | 0.1 | 1.1 |  |  |
| **max** | 1.00 | 8.3 | 2.1 | 217.3 | 79.9 | 5.5 | 0.8 | 2.2 |  |  |

**Table S8. Calculations of the parameter *p* based on the estimated value of *pT*_0_, using two assumptions for *T*_0_: when *T*_0_ corresponds to the total or 5% of the CD4^+^ baseline count. These estimates are for fittings assuming *f*=0.05.**

|  | **Estimated log_10_(*pT*_0_)** log_10_(cell/ml/day) | ***T*_0_=CD4^+^ baseline count** | | ***T*_0_=5% of CD4^+^ baseline count** | | **Measured baseline viral load** (copies/ml) |
| --- | --- | --- | --- | --- | --- | --- |
|  |  | ***T*_0_** (cells/ml) | ***p*** (1/day) | ***T*_0_** (cells/ml) | ***p*** (1/day) |  |
| **#20** | 8.3 | 312500 | 607 | 15625 | 12140 | 2576 |
| **#22** | 7.6 | 934500 | 39 | 46725 | 779 | 522 |
| **#23** | 8.3 | 405500 | 513 | 20275 | 10268 | 17719 |
| **#24** | 7.8 | 228500 | 302 | 11425 | 6035 | 5370 |
| **#25** | 8.3 | 247500 | 766 | 12375 | 15319 | 27090 |
| **#27** | 7.2 | 1189500 | 13 | 59475 | 264 | 170 |
| **Median** | 8.1 | 359000 | 408 | 17950 | 8151 |  |
| **min** | 7.2 | 228500 | 13 | 11425 | 264 |  |
| **max** | 8.3 | 1189500 | 766 | 59475 | 15319 |  |

**Table S9. Parameters estimates of the models to each individual viral load data with the lowest BIC values in Table 2, but assuming that *f*=1 (i.e. there is not abortive infection).**

|  | **%*V_s_*(0)** | **log_10_(*pT*_0_)** | ***K_ds_*** | ***K_dr_*** | ***γ*** | **log_10_(*K*)** | ***m*** | ***ω*** |
| --- | --- | --- | --- | --- | --- | --- | --- | --- |
|  | (-) | log_10_(1/ml/day) | (μg/ml) | (μg/ml) | (1/day) | log_10_(1/ml) | (1/day) | (-) |
| **#20** | - | 7.0 | 1.0 | - | 75.9 | 4.5 | 0.8 | 2.5 |
| **#22** | - | 6.3 | 0.4 | - | 23.2 | 3.9 | 0.1 | 1.2 |
| **#23** | 0.85 | 7.0 | 2.1 | 28.9 | 27.7 | 5.4 | 0.3 | 1.3 |
| **#24** | 0.95 | 6.5 | 0.7 | 227.2 | 60.4 | 4.8 | 0.2 | 1.2 |
| **#25** | 0.99 | 7.0 | 0.4 | 130.1 | 55.2 | 5.5 | 0.2 | 1.2 |
| **#27** | - | 5.9 | 15.3 | - | 24.1 | 3.3 | 0.2 | 1.1 |
| **Median** | 0.95 | 8.1 | 0.9 | 91.5 | 43.5 | 4.7 | 0.2 | 1.2 |
| **min** | 0.85 | 7.2 | 0.29 | 28.9 | 23.0 | 3.3 | 0.1 | 1.1 |
| **max** | 0.99 | 8.3 | 15.9 | 217.3 | 75.9 | 5.5 | 0.8 | 2.5 |

**Table S10. Calculations of the parameter *p* based on the estimated value of *pT*_0_, using two assumptions for *T*_0_: when *T*_0_ corresponds to the total or 5% of the CD4^+^ baseline count. These estimates are for fittings assuming *f*=1.**

|  | **Estimated log_10_(*pT*_0_)** log_10_(cell/ml/day) | ***T*_0_=CD4^+^ baseline count** | | ***T*_0_=5% of CD4^+^ baseline count** | |
| --- | --- | --- | --- | --- | --- |
|  |  | ***T*_0_** (cells/ml) | ***p*** (1/day) | ***T*_0_** (cells/ml) | ***p*** (1/day) |
| **#20** | 7.0 | 312500 | 30 | 15625 | 607 |
| **#22** | 6.3 | 934500 | 2 | 46725 | 39 |
| **#23** | 7.0 | 405500 | 26 | 20275 | 514 |
| **#24** | 6.5 | 228500 | 15 | 11425 | 305 |
| **#25** | 7.0 | 247500 | 42 | 12375 | 830 |
| **#27** | 5.9 | 1189500 | 1 | 59475 | 13 |
| **Median** | 6.8 | 359000 | 21 | 17950 | 409 |
| **min** | 5.9 | 228500 | 1 | 11425 | 13 |
| **max** | 7.0 | 1189500 | 42 | 59475 | 830 |

**Table S11. Comparing best model with respect to the case when *ω*=1 (constant death rate of infected cells).**

|  | **BIC (PLCM2)** | |
| --- | --- | --- |
|  | ***ω* >1** | ***ω* =1** |
| **#20** | -48.8 | -37.34 |
| **#22** | -47.4 | -27.99 |
| **#23** | -42.1 | -24.05 |
| **#24** | -68.7 | -29.42 |
| **#25** | -34.6 | -14.90 |
| **#27** | -36.3 | -29.34 |

**Table S12.** BIC values from the sum of squares error to the best fits of each model to the 10 viral load profiles with added noise from each subject’s viral load data. In bold, the lowest BIC of each row.

|  | **BIC** | | | | | |
| --- | --- | --- | --- | --- | --- | --- |
|  | **DNM** | | **PSCM** | | **PLCM** | |
| **ID** | **1 Viral pop.** | **2 Viral pops.** | **1 Viral pop.** | **2 Viral pops.** | **1 Viral pop.** | **2 Viral pops.** |
| **#20** | -24.5 | -23.6 | 18.8 | 19.7 | **-33.9** | -18.7 |
| **#22** | 0.6 | 3.4 | 26.9 | 22.1 | **-18.9** | -17.9 |
| **#23** | -23.2 | -20.8 | 24.6 | 25.3 | -24.9 | **-31.3** |
| **#24** | -21.3 | -21.8 | 27.5 | 29.3 | -27.6 | **-27.8** |
| **#25** | -21.4 | -18.8 | 28.9 | 34.8 | -19.2 | **-23.0** |
| **#27** | -17.4 | -14.8 | 25.5 | 24.1 | **-20.5** | -15.9 |

DNM: Delayed neutralization model.

PSCM: Phagocytosis-based saturated clearance model.

PLCM: Phagocytosis-based logistic clearance model.
